# Administration of an AAV vector coding for a P2X7-blocking nanobody-based biologic ameliorates colitis in mice

**DOI:** 10.1186/s12951-023-02285-4

**Published:** 2024-01-11

**Authors:** Catalina Abad, Mélanie Demeules, Charlotte Guillou, Henri Gondé, Rachid Zoubairi, Yossan-Var Tan, Carolina Pinto-Espinoza, Waldemar Schäfer, Anna Marei Mann, Valérie Vouret-Craviari, Friedrich Koch-Nolte, Sahil Adriouch

**Affiliations:** 1 Univ Rouen Normandie, INSERM, U1234, Pathophysiology Autoimmunity and Immunotherapy (PANTHER), Normandie Univ, 76000 Rouen, France; 2 Institute of Immunology, University Medical Center Hamburg-Eppendorf, Hamburg, Germany; 3 CNRS, INSERM, IRCAN, Université Côte d’Azur, Nice, France; 4 FHU OncoAge, Nice, France; 5 Faculty of Medicine and Pharmacy, INSERM U1234 – PANTHER Lab, 22 Boulevard Gambetta, CS 76183, University of Rouen, 76000 Rouen, France

**Keywords:** Adeno-associated virus, Nanobodies (VHH), P2X7, Animal models, Colitis

## Abstract

**Background:**

The pro-inflammatory ATP-gated P2X7 receptor is widely expressed by immune and non-immune cells. Nanobodies targeting P2X7, with potentiating or antagonistic effects, have been developed. Adeno-associated virus (AAV)-mediated gene transfer represents an efficient approach to achieve long-term in vivo expression of selected nanobody-based biologics. This approach (AAVnano) was used to validate the relevance of P2X7 as a target in dextran sodium sulfate (DSS)-induced colitis in mice.

**Results:**

Mice received an intramuscular injection of AAV vectors coding for potentiating (14D5-dimHLE) or antagonistic (13A7-Fc) nanobody-based biologics targeting P2X7. Long-term modulation of P2X7 activity was evaluated ex vivo from blood samples. Colitis was induced with DSS in mice injected with AAV vectors coding for nanobody-based biologics. Severity of colitis, colon histopathology and expression of chemokines and cytokines were determined to evaluate the impact of P2X7 modulation. A single injection of an AAV vector coding for 13A7-Fc or 14D5-dimHLE efficiently modulated P2X7 function in vivo from day 15 up to day 120 post-injection in a dose-dependent manner. An AAV vector coding for 13A7-Fc significantly ameliorated DSS-induced colitis and significantly reduced immune cell infiltration and expression of chemokines and proinflammatory cytokines in colonic tissue.

**Conclusions:**

We have demonstrated the validity of AAVnano methodology to modulate P2X7 functions in vivo. Applying this methodological approach to a DSS-induced colitis model, we have shown that P2X7 blockade reduces inflammation and disease severity. Hence, this study confirms the importance of P2X7 as a pharmacological target and suggests the use of nanobody-based biologics as potential therapeutics in inflammatory bowel disease.

**Graphical Abstract:**

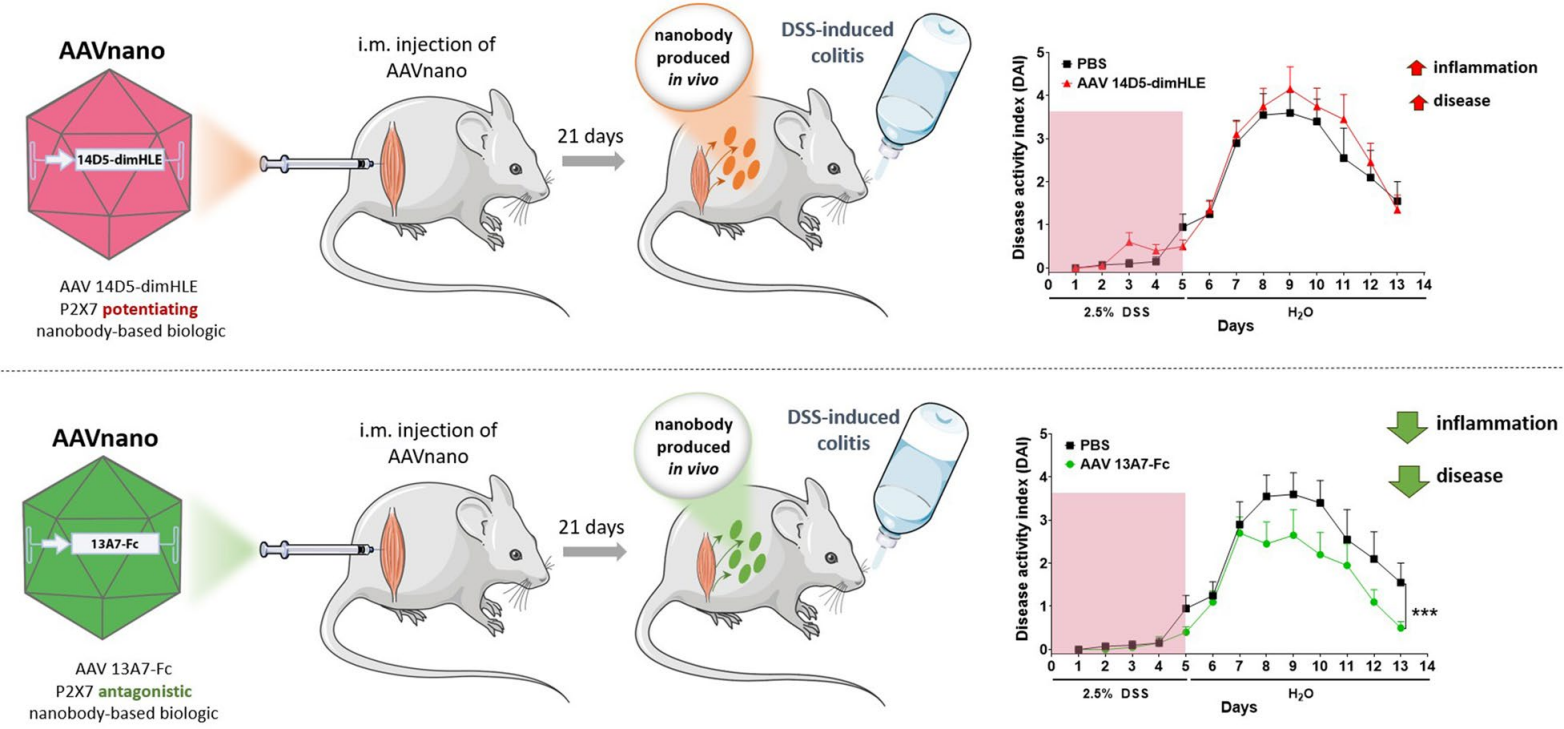

**Supplementary Information:**

The online version contains supplementary material available at 10.1186/s12951-023-02285-4.

## Background

Purine nucleotides released into the extracellular compartment, such as extracellular ATP (eATP), can act as potent signalling molecules that regulate several physiological processes, including immune responses. Whereas eATP levels remain low in homeostatic conditions, eATP concentrations rapidly increase in situations of cell stress or cell damage. Then, eATP can act as a danger associated molecular pattern (DAMP) engaging an immune response. Indeed, eATP has recently emerged as a key signalling molecule mediating different aspects of innate and adaptive immunity [1–3]. Notably, eATP acts through the purinergic P2X7 receptor, a ligand-gated ion channel of the purinergic type 2 receptor (P2R) family [4]. P2X7 is widely expressed by lymphoid and myeloid immune cells [5]. In murine T lymphocytes, P2X7 activation induces metalloprotease-mediated shedding of CD62L and CD27 and leads to regulatory T cell (Treg) death [2, 6–11]. In myeloid cells such as macrophages and dendritic cells, gating of P2X7 drives activation of the NLRP3 inflammasome, caspase-1 and gasdermin D, and the subsequent maturation and release of mature IL-1β and IL-18 proinflammatory cytokines [12–15].

Ample evidence suggests a role of eATP in the pathogenesis of inflammatory diseases including inflammatory bowel disease (IBD) [16, 17]. P2X7 is expressed in immune and non-immune cells of the gastrointestinal tract [11, 18–21]. Up-regulated P2X7 expression has been reported in the colon epithelium and in the lamina propria (macrophages and dendritic cells) of Crohn’s disease patients [22]. Similarly, up-regulated expression of P2X7 has been reported in rats subjected to an acute model of dextran sodium sulfate (DSS)-induced colitis [23]. Conversely, P2X7-deficient mice exhibit reduced mucosal inflammation in both, DSS and trinitrobenzene sulfonic acid (TNBS)-induced models of colitis [22, 24, 25]. These findings agree with the purported proinflammatory role of P2X7, and support its role in the development of intestinal inflammation. Moreover, these findings suggest that P2X7 may constitute a therapeutic target in inflammatory diseases such as IBD. Despite the development of several small-molecule P2X7 inhibitors [26], their use may be hampered by their low specificity, their side effects, and their short half-life in vivo due to their metabolism into inactive by-products.

Antibodies constitute important tools for experimental research and therapy. Most antibodies are composed of two heavy and two light chains and both chains contribute to the antigen-binding site. Remarkably, llamas and other camelids can also produce antibodies composed of only heavy chains [27]. The single antigen-binding domain of these antibodies is designated V_H_H or, when produced as a recombinant protein, nanobody (Nb) or single domain antibody. Nanobodies that block (13A7) or potentiate (14D5) gating of the P2X7 ion channel have been developed [28, 29]. The efficacy of these nanobodies in vivo has been demonstrated, since systemic injection of recombinant 13A7-based biologics in mice blocked P2X7 activation in T cells and macrophages and ameliorated experimental glomerulonephritis and allergic contact dermatitis [28]. Yet, injections every three days were necessary to maintain effective concentrations of the nanobody in blood. Here, we report an alternative adeno-associated virus (AAV)-based methodology as a delivery vector for the in vivo gene transfer of DNA sequences coding for selected nanobody-based biologics. With this strategy, termed AAVnano, we screened directly in vivo the best nanobody-based biologic candidates, while maintaining an ideal bioavailability over time, with the aim to validate P2X7 as a potential pharmacological target. Using the AAVnano methodological approach, we were able to ensure the continuous presence of effective concentrations of the nanobody-based biologics evaluated in vivo, even in the long-term, up to at least 120 days post AAV administration. We first identified and characterized AAV vectors coding for nanobody-based biologics able to either block or potentiate P2X7 function. One AAV was selected for its capacity to potently and durably inhibit P2X7 in vivo (i.e., coding for 13A7-Fc biologic), and one for its capacity to potentiate P2X7 functions in vivo (i.e., coding for 14D5-dimHLE biologic). This methodological approach offers the unique opportunity to directly modulate P2X7 functions in vivo, in different pathophysiological situations, and in any given mouse genetic background (contrary to genetically-manipulated animals). Also, there is no need to perform repeated small-molecule injections for which pharmacokinetic profile and bioavailability are not always sufficiently characterized. In addition, this methodological approach allowed not only to inhibit P2X7 (i.e., using AAV-13A7-Fc), a frequent aim of published studies, but also to potentiate its function (i.e., using AAV-14D5-dimHLE) which may aggravate the disease, thus confirming indirectly the implication of P2X7 in a given pathophysiological process. More specifically, we aimed to evaluate P2X7 contribution to the pathological inflammatory condition induced by DSS administration, as a model of inflammatory colitis. In this model, we demonstrated that a single injection of AAV-13A7-Fc significantly reduced DSS-induced colitis, and inhibited immune cell infiltration and production of inflammatory cytokines. In conclusion, our data validated the AAVnano methodological approach to study P2X7 contribution in vivo and, more importantly, confirmed the relevance of P2X7 as a pharmacological target. Additionally, our study suggests that a therapeutic strategy using nanobody-based biologics to target P2X7 may be beneficial for the treatment of IBD, and potentially other chronic inflammatory diseases.

## Results

### AAVnano methodology to evaluate in vivo different nanobody-based biologics designed to modulate P2X7 functions

**Fig. 1:**
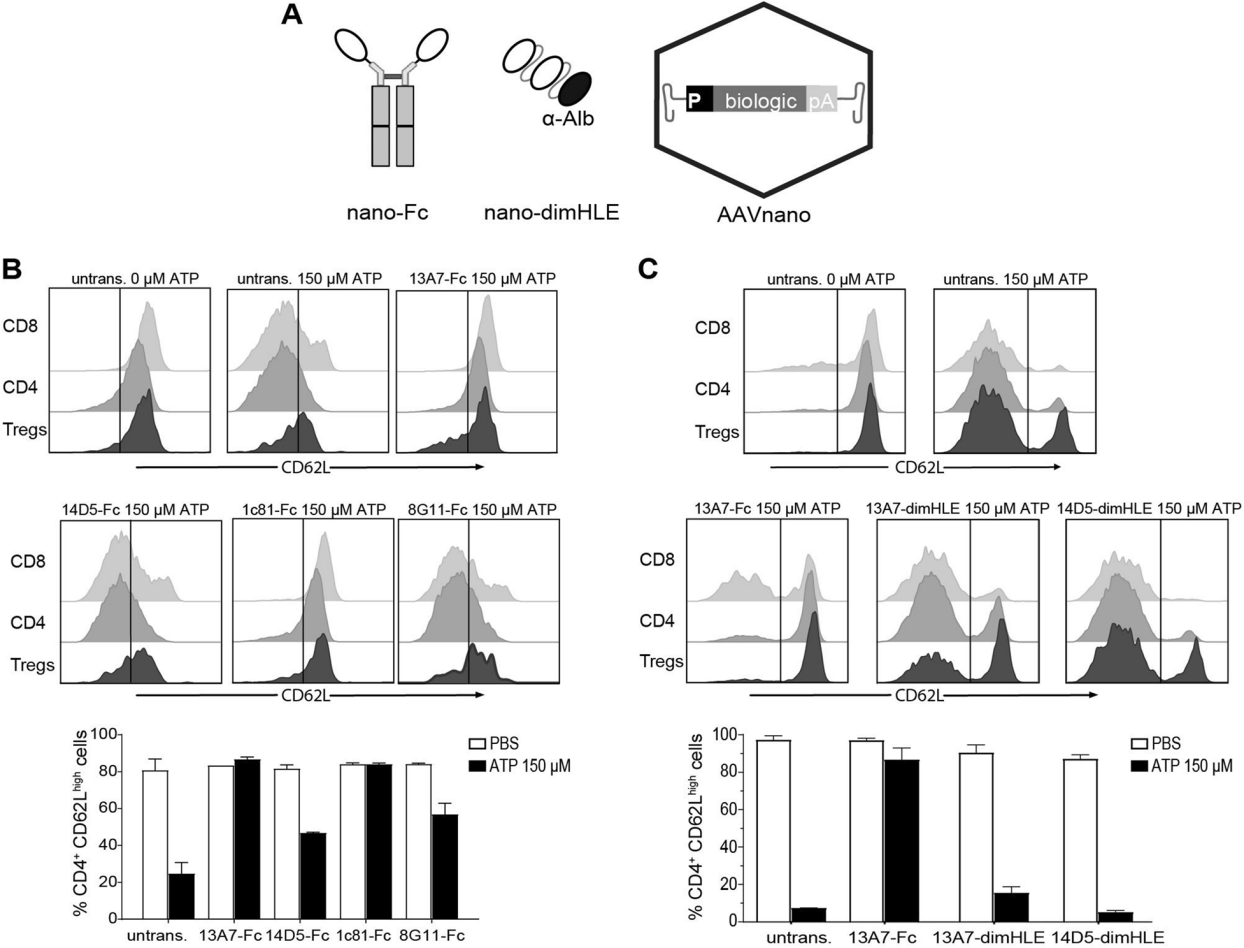
Comparison of different AAVnano vectors and their potential to modulate P2X7 function in vivo after i.m. administration. Mice (n = 3) were injected with Nb-encoding AAV vectors in the gastrocnemius muscles at day 0. **A** Scheme of the nanobody-based biologics used in our study and the AAVnano vectors coding for these constructs. Two formats were evaluated, a nanobody-Fc fusion protein, termed nano-Fc, where the Fc region of mouse IgG1 carries the “LSF” mutations T252L, T254S, T256F to confer increased half-life in vivo, or a nanobody dimer (dim) fused to a third anti-albumin (Alb8) nanobody, termed nano-dimHLE, that also confers half-life extension (HLE) in vivo. **B**, **C** AAVnano vectors coding for the indicated nanobody-based biologics were injected i.m. at a dose of 10^11^ vg/mouse. Control mice were not transduced with AAV (untransd.). Blood samples were collected 15 (**B**) or 17 (**C**) days post i.m. injections and the functional activity of P2X7 on the surface of T cell subsets was evaluated after incubation with 150 µM ATP. Flow cytometry profiles of CD62L expression and percentages of CD4^+^CD62L^high^ cells in the gated CD8^+^, CD4^+^CD25^−^, or CD4^+^CD25^+^ (Tregs) subsets are shown. One representative experiment out of at least two is shown with n = 3 mice per experiment

Different nanobodies targeting P2X7 were previously generated that exhibited an ability to block or to potentiate P2X7 functions [28]. We selected three nanobody clones that previously demonstrated an antagonist effect in vitro (*i.e.,* clones 13A7, 1c81 and, 8G11) and one clone with a potentiating activity (*i.e.,* clone 14D5); patent WO2013178783A1 [30]. As a first step, we aimed to select among these clones the nanobody-based biologic offering the highest capacity to inhibit, or conversely to potentiate, P2X7 functions in vivo. Nanobodies display a limited in vivo persistence due to their small size and the lack of an Fc-region that mediates half-life extension through the neonatal Fc receptor (FcRn). We thus generated two types of constructs to increase their half-life and their avidity for the target. In one construct, we fused the selected nanobody to the hinge and Fc regions of a variant of mouse IgG1 carrying the “LSF” mutations (T252L, T254S, T256F), which were shown to confer a higher affinity to FcRn [28, 31]. In the second construct, we dimerised the selected nanobody and further fused the construct to an albumin-specific nanobody (Alb8), conferring half-life extension by binding to albumin [28, 31]. The corresponding genetic sequences coding for these two types of constructs, termed respectively nano-Fc or nano-dimHLE, were used to generate recombinant AAV vectors coding for the nanobody-based biologics of interest (Fig. 1A).

In a first screening phase, we evaluated in vivo four different AAVs, each coding for a different nano-Fc construct targeting P2X7 (*i.e.,* 13A7-Fc, 14D5-Fc, 1c81-Fc and 8G11-Fc). For that, AAVs were administered intramuscularly (i.m.) at a dose of 10^11^ vg/mouse, and the functional consequences of the nano-Fc biologics produced in vivo were evaluated. As a robust and sensitive readout of P2X7 activity, we used its known effect on metalloprotease-mediated release of CD62L from the surface of peripheral blood T cells [6, 8]. Blood samples were collected 2 weeks post AAV injection, treated with ATP ex vivo, and P2X7-dependent shedding of CD62L was evaluated by flow cytometry in CD8^+^, CD4^+^ and Treg (CD4^+^CD25^+^) cell subsets. The results indicate that AAVs coding for 13A7-Fc or 1c81-Fc efficiently blocked P2X7 activity, while the AAV coding for 8G11-Fc was associated with partial inhibition (Fig. 1B).

Unexpectedly, while nanobody 14D5 was formerly found to potentiate P2X7 receptor activity [28], the 14D5-Fc construct evaluated here did not show a potentiating effect, but instead showed a weak antagonistic activity possibly due to steric hindrance or to 14D5-Fc induced P2X7-endocytosis (Fig. 1B).

**Fig. 2:**
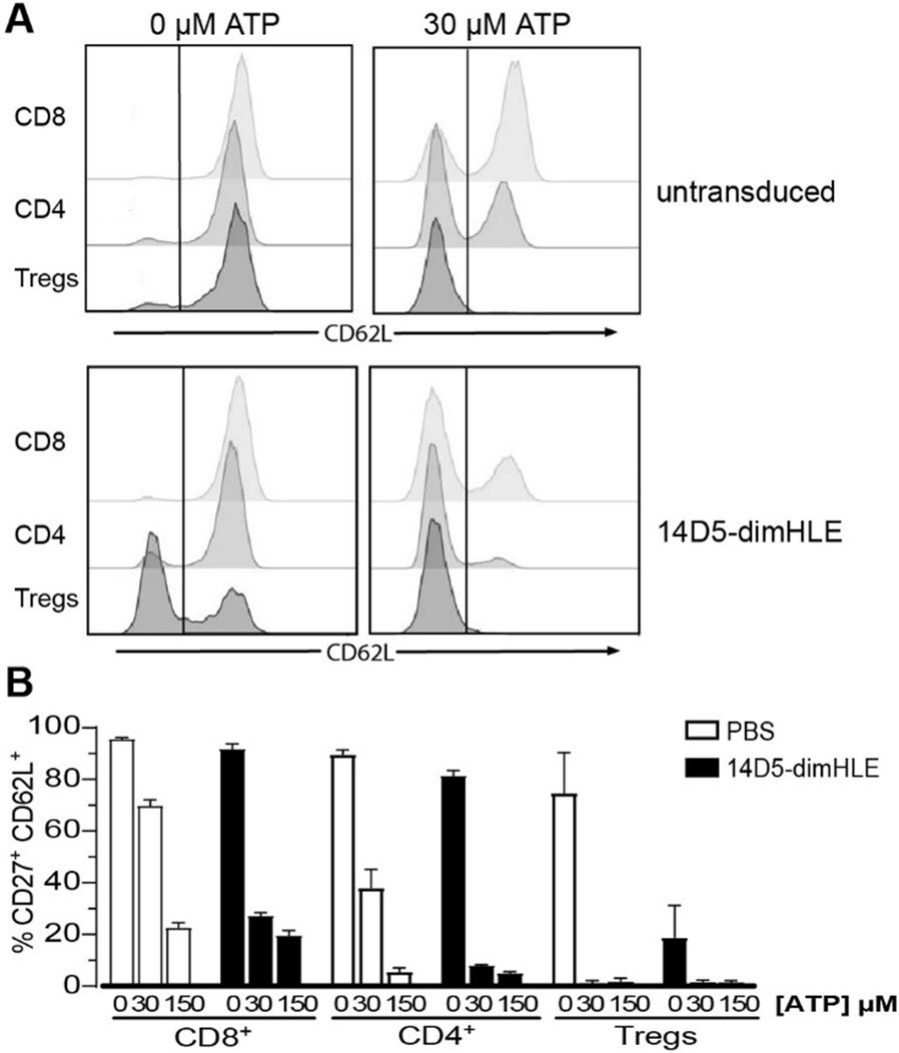
Evaluation of the potentiating effect of AAV-14D5-dimHLE vector on the activity of P2X7 following i.m. administration. AAV-14D5-dimHLE vector was injected i.m. at a dose of 10^11^ vg/mouse. Blood cells were collected 21 days later and the functional activity of P2X7 on the surface of T cell subsets was evaluated after incubation with 30 µM or 150 µM ATP. Flow cytometry profiles of CD62L expression (**A**) and percentages (**B**) of CD4^+^CD27^+^CD62L^high^ cells in the gated CD8^+^, CD4^+^CD25^−^, or CD4^+^CD25^+^ (Tregs) subsets are shown. One representative experiment out of at least two is shown with n = 3 mice per experiment

We next evaluated whether a construct based on the nano-dimHLE format could better preserve the potentiating effect observed in vitro with the original 14D5 nanobody. We thus generated an AAV vector coding for 14D5-dimHLE as well as, for comparison, a vector coding for 13A7-dimHLE. Mice were injected with the different AAV vectors and blood samples were collected 2 weeks later for evaluation of P2X7 function ex vivo in T cells. The results show that the AAV vector coding for the 13A7-Fc construct blocked P2X7 function more extensively than the new one coding for 13A7-dimHLE, suggesting an influence of format on potency in vivo. The data also show that the AAV vector coding for 14D5-dimHLE indeed exerted a potentiating effect on P2X7 activity, as initially aimed (Fig. 1C). However, as the chosen dose of 150 µM ATP already represented a near saturating dose in our sensitive assay, the potentiating effect of 14D5-dimHLE was barely visible in this condition. To better evaluate this point, we repeated the experiment to include a lower dose of 30 µM ATP and analyzed the shedding of CD62L and CD27 in CD8^+^, CD4^+^ and CD4^+^CD25^+^ T cell subsets. As expected, the potentiating effect was now evident in all analyzed T cell subsets, including in CD8^+^ T cells that express lower levels of P2X7 [32, 33] and are less sensitive than CD4^+^ subsets (Fig. 2A, B). Thus, these data show that P2X7 activation in vivo was facilitated by 14D5-dimHLE at low ATP concentrations after i.m. administration of the corresponding AAV vector, AAV-14D5-dimHLE.

Collectively, based on these results we selected AAV-13A7-Fc as the most potent AAV vector to suppress P2X7 function in vivo and AAV-14D5-dimHLE as the most potent AAV vector to potentiate P2X7 at low ATP concentrations.

### Evaluation of the AAV dose required to block P2X7 function in vivo

**Fig. 3:**
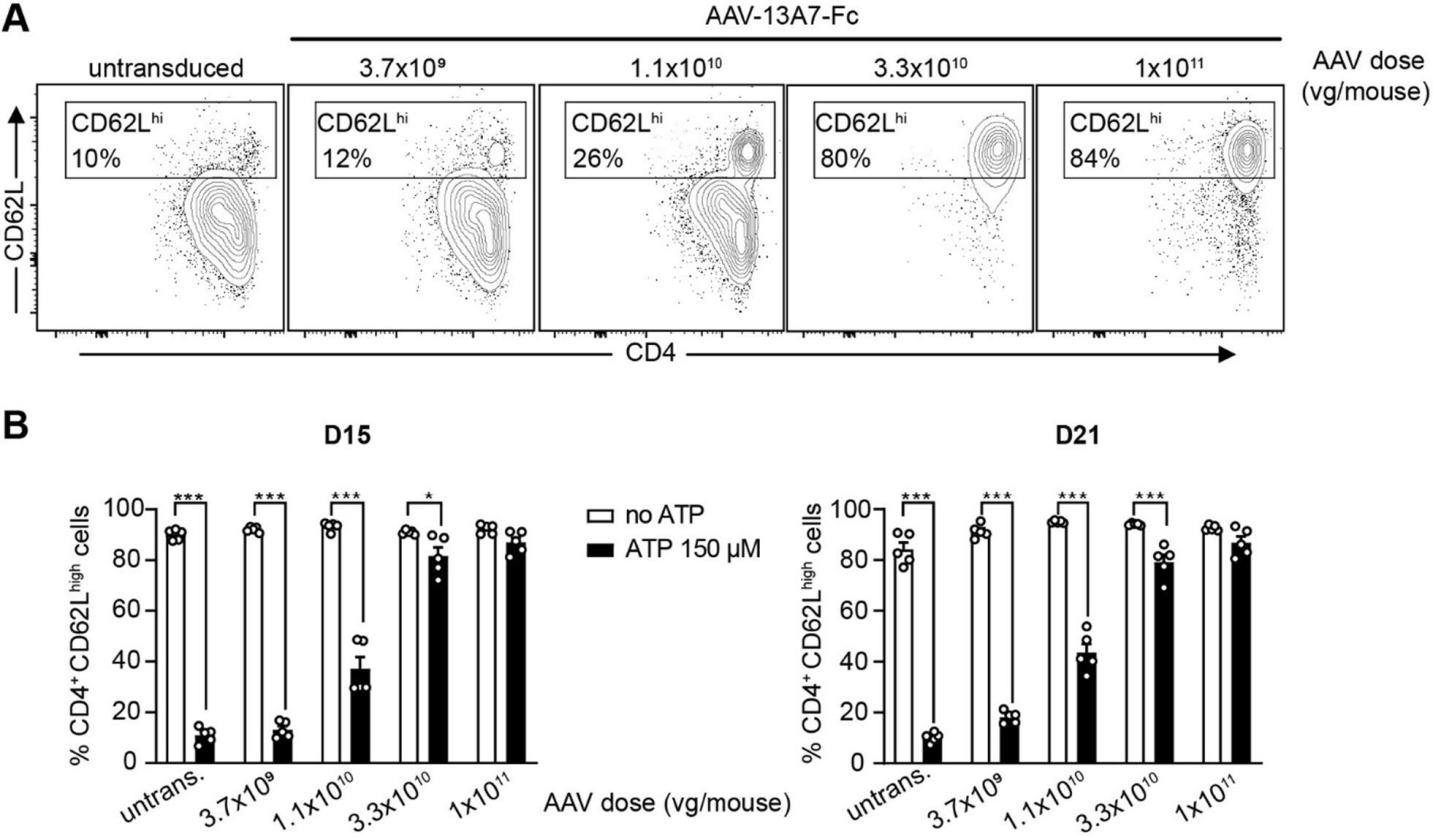
Evaluation of the blocking effect of AAV-13A7-Fc vector on the activity of P2X7 following i.m. administration of different doses of AAVnano vector. AAV-13A7-Fc vector was injected i.m. at the indicated AAVnano dose and blood cells were collected 15 and 21 days later to evaluate the functional activity of P2X7 on the surface of CD4^+^ T cells upon incubation with 150 µM ATP for 15 min. **A** Representative flow cytometry panels illustrating the ATP-induced P2X7-dependant CD62L shedding within gated CD4^+^ T cells, 15 days post-AAV injection (numbers correspond to the percentages of CD4^+^ CD62L^high^ cells). The percentages obtained in the absence of ATP is 86% (not shown). **B** Percentages of CD4^+^ CD62L^high^ cells in each experimental condition, from blood cells collected 15 or 21 days post-AAV injection, after treatment ex vivo with 150 µM ATP for 15 min. Results represent mean values ± SEM, with n = 5 mice per group. *p < 0.05, ***p < 0.001 two-way ANOVA

Next, we evaluated the dose–response of our AAVnano methodology. For that, we focused here on the AAV-13A7-Fc vector as complete inhibition of P2X7 function requires a saturating dose of nanobody-based biologics, while partial versus full potentiation is more difficult to distinguish. With that aim, different doses ranging from 3.7 × 10^9^ vg to 1 × 10^11^ vg per mouse of AAV-13A7-Fc were injected i.m. and blood samples were collected 2 and 3 weeks later to evaluate ATP-induced shedding of CD62L from the surface of CD4^+^ T cells as previously described. The results demonstrate that almost complete blockade of P2X7 function can be achieved at vector doses above 3.3 × 10^10^ vg/mouse (Fig. 3). To confirm and extend these results, a similar experiment was performed at a later time point (12 weeks post-AAV injection) to evaluate the durability of P2X7 blockade in vivo*.* Here we included higher doses of ATP and extended the analyses to CD4^+^ and CD8^+^ T cell subsets. The results confirm the initial dose–response profile, with a near complete P2X7-blockade at AAV-doses above 1 × 10^10^ vg/mouse (Additional file 1: Fig. S1, S2). Remarkably, we observed that both, the CD8^+^ and CD4^+^ T cells collected from AAV-13A7-Fc injected mice were resistant to very high ATP concentrations (*i.e.,* up to 600 µM), confirming the high level of P2X7-inhibition obtained with our AAVnano methodology. Based on the results of this dose–response study, we chose to inject a saturating dose of 1 × 10^11^ vg per mouse in all subsequent studies. Importantly, we confirmed the absence of any obvious signs of toxicity or morbidity upon injection of our AAV at this dose, in agreement with the safety profile of AAV vectors (Additional file 1: Fig. S3).

### Evaluation of the kinetics of production in vivo of nanobody-based biologics and of their P2X7-modulating effects following a single injection of AAVnano

**Fig. 4:**
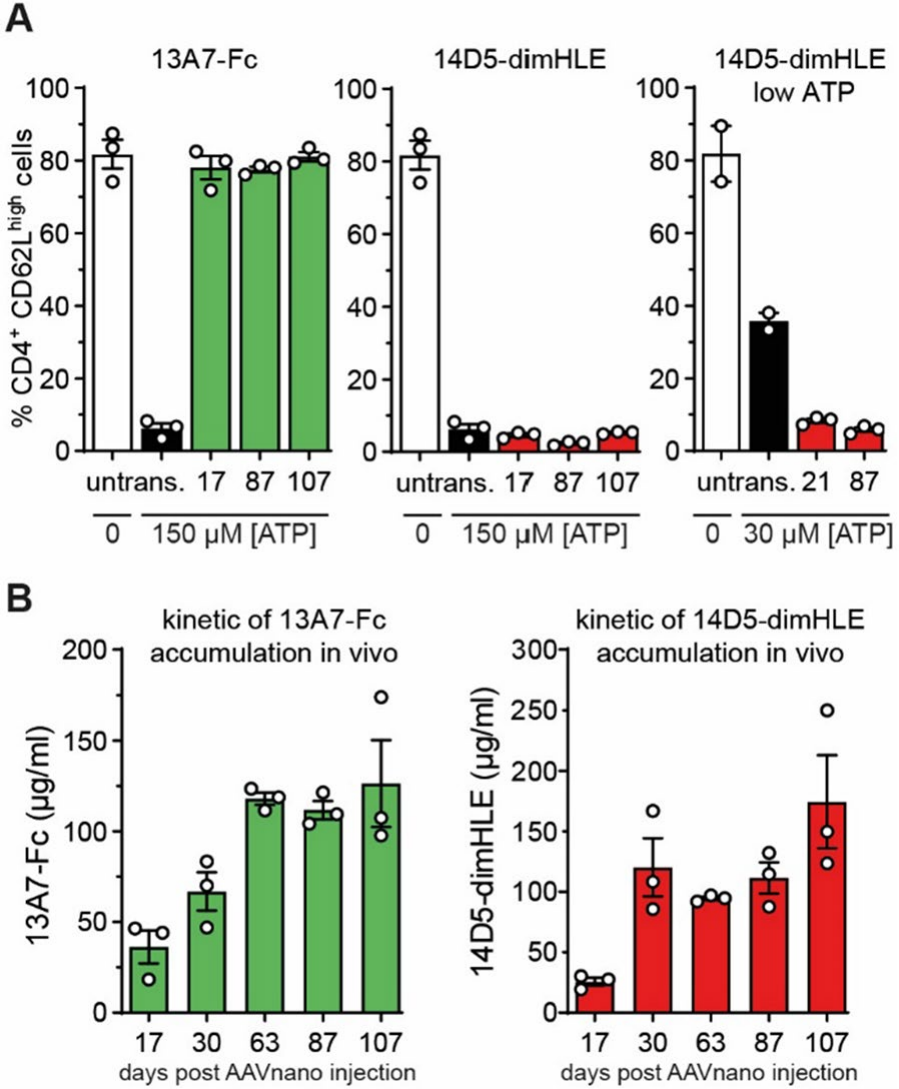
Evaluation of AAV-13A7-Fc blocking and of AAV-14D5-dimHLE potentiating vectors on the activity of P2X7 over time and evaluation of the kinetics of production of nanobody-based biologics in vivo after a single i.m. injection of each AAVnano vector. AAV-13A7-Fc or AAV-14D5-dimHLE vectors were injected i.m. at a dose of 10^11^ vg/mouse and blood cells or sera were collected at the indicated time points. **A** Blood cells were incubated with 150 µM or 30 µM ATP, as indicated, and the percentages of CD4^+^CD62L^high^ cells were determined by flow cytometry as an evaluation of P2X7 activity on the surface of the gated CD4^+^ T cells. The first two bars in each graph correspond, respectively, to negative and positive control cells collected from untransduced control mice treated or not with the indicated concentration of ATP. Results represent mean values ± SEM, with n = 2–3 mice per group. **B** The concentration of unbound 13A7-Fc and 14D5-dimHLE nanobody-based biologics in the sera collected at each indicated time point was titrated by flow cytometry using a P2X7-expressing cell line and standard curves obtained with the same recombinant biologics produced in vitro. Results represent mean values ± SEM, with n = 3 mice per group

To better characterize our AAVnano methodology, we further determined the effects of 13A7-Fc and 14D5-dimHLE over time. For that, the corresponding AAV vectors were injected i.m at a dose of 10^11^ vg/mouse, and blood samples were collected at different time points to evaluate ex vivo the effect of each construct on the activity of P2X7 on the surface of circulating T cells. The blocking potential of 13A7-Fc nanobodies was evaluated and compared to ATP-untreated T cells collected from untransduced control mice. The results confirm a complete blockade of P2X7 at all time points analyzed, suggesting a durable effect of the antagonistic nanobody-based biologics in vivo (Fig. 4A, left panel, and Additional file 1: Fig. S4). For further confirmation of the potent blocking effect of AAV-delivered 13A7-Fc on P2X7 function, we also performed comparisons with cells obtained from P2X7^−/−^ mice. For that, mice were euthanized 120 days post AAV-injection and splenocytes were collected. The cells were treated ex vivo with 150 µM ATP and P2X7 activity was evaluated in these cells and compared with splenocytes collected from P2X7^−/−^ mice. The results demonstrate that the functional inhibition of P2X7 obtained 120 days post AAV-13A7-Fc was similar to that observed with cells from P2X7-deficient mice (Additional file 1: Fig. S5).

We also evaluated the potentiating effect of 14D5-dimHLE on P2X7 function over time. At some time points (*i.e.,* 17, 87, and 107 days post AAV-injection), cells were treated with a dose of 150 µM ATP, while for some time points (*i.e.,* 21 and 87 days post AAV-injection) cells were also treated with a lower dose of 30 µM ATP to evaluate the increase in sensitivity to lower ATP concentrations. The results show that the effect of 14D5-dimHLE on the activity of P2X7 was barely visible in the experimental setting using 150 µM ATP (Fig. 4A, middle panel, and Additional file 1: Fig. S4). However, a potentiating effect was clearly evidenced at the lower dose of 30 µM ATP (Fig. 4A, right panel, and Additional file 1: Fig. S4). This confirms previous data, suggesting that 14D5-dimHLE mediates a 3- to sixfold increased sensitivity to low ATP concentrations.

Next, we evaluated the kinetics of production of the nanobody-based biologics in vivo after a single administration of the corresponding AAV vector. For that, 10^11^ vg/mouse of AAV-13A7-Fc or AAV-14D5-dimHLE were injected i.m. and sera were collected over time for more than 100 days. The concentration of unbound nanobody-based biologics in these samples was evaluated using a flow cytometric assay. For that, a P2X7-expressing cell line was used to capture the nanobody-based biologics contained in the diluted serum, and the respective nanobody concentration was determined by comparison with a standard titration curve. The results reveal that nanobody-based biologics reached concentrations of ~ 30 µg/ml at day 17 post AAV injections. Concentrations subsequently increased over time to reach a plateau at ~ 100 µg/ml between days 30 and 60 that was maintained over time until experiment termination (Fig. 4B). These data support the notion that AAVnano methodology leads to a continuous production of nanobody-based biologics for several months in vivo, with relatively constant pharmacokinetics, contrasting with that usually obtained using repeated injections of recombinant biologics.

Importantly, no conspicuous changes were observed in the frequency of T cell subsets over time in the different AAV-injected groups, suggesting that the nanobody-based biologics evaluated here do not induce depletion of the target cells (not shown). Of note, even if we only used a single assay to evaluate P2X7 activity (*i.e.,* a sensitive assay based on the shedding of CD27/CD62L in T cells), we previously demonstrated that the same constructs similarly modulated other hallmarks associated with P2X7 functions, in lymphoid as well as myeloid cells, such as membrane depolarisation, calcium influx, pore formation, NLRP3 oligomerisation, or IL-1β release [28, 30, 31]. Furthermore, we recently found that AAV-13A7-dimHLE and AAV-13A7-Fc also inhibited P2X7-dependent pore formation and IL-1β release in microglial cells in vivo across the blood–brain-barrier [34].

Hence, these data collectively indicate that nanobody-based biologics produced in vivo following AAV-mediated gene transfer are well tolerated*,* durably expressed, and potently modulate (*i.e.,* block or potentiate) P2X7 functions in vivo.

### AAV-13A7-Fc ameliorates the clinical and histopathological features of DSS-induced colitis

**Fig. 5:**
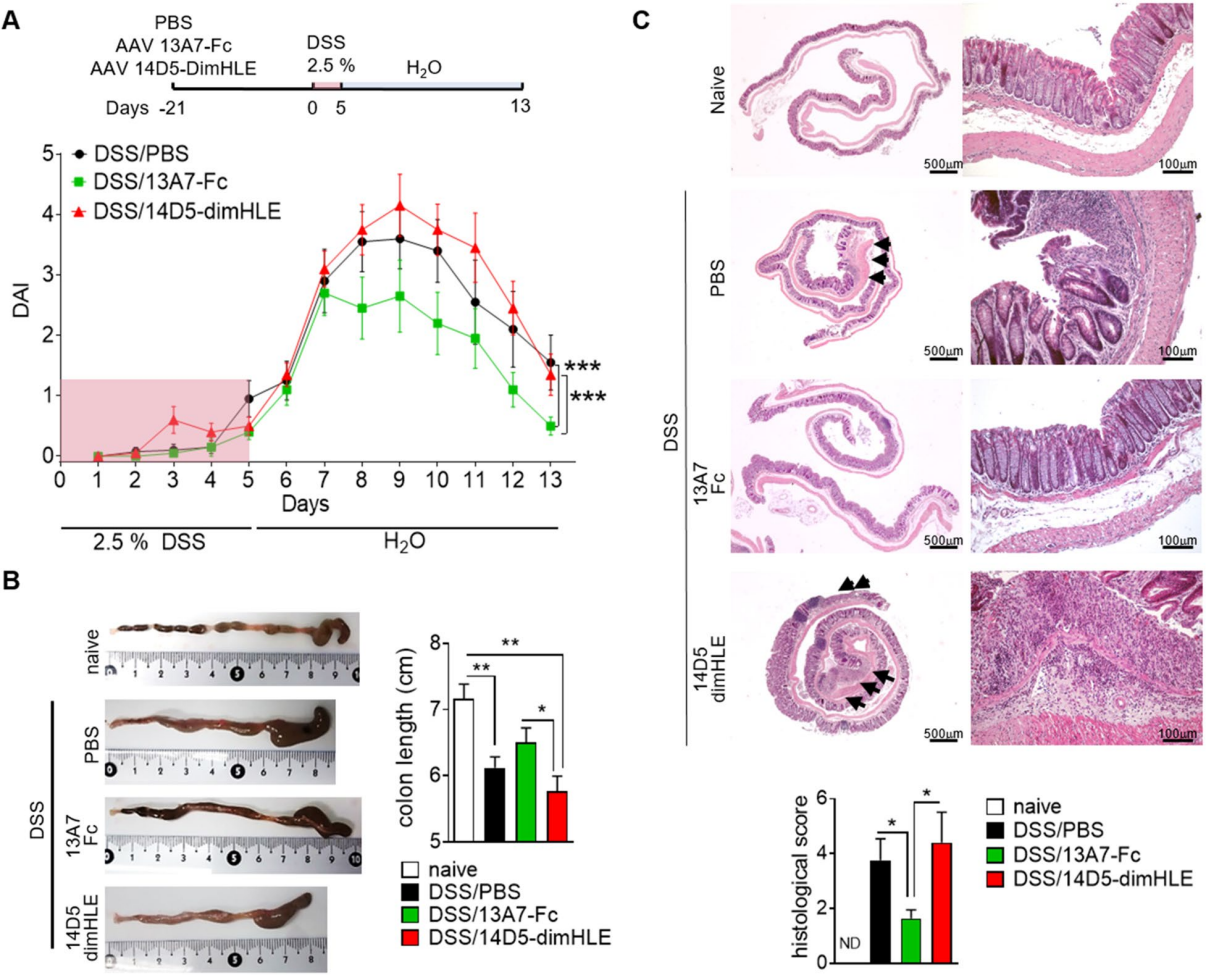
AAV-13A7-Fc administration ameliorated acute DSS-induced colitis. Mice (n = 10) received 10^11^ vg of AAVnano coding for 13A7-Fc or 14D5-dimHLE and three weeks later they were given 2.5% DSS in drinking water for five days. Mice were switched to regular water for eight additional days and colons were collected at the end of the study. **A** Disease activity score (DAI). **B** Colon macroscopic appearance. **C** Histopathological analysis of colon stained with hematoxylin and eosin staining. Mean ± SEM are shown. Statistical comparison Two-way ANOVA (**A**) or one-way ANOVA (**B**, **C**) was performed with *p < 0.05 and **p < 0.01. One representative experiment out of three is shown

P2X7 contributes to inflammatory response by activating the NLRP3 inflammasome that leads to caspase-1 activation and IL-1β and IL-18 maturation and release. In order to investigate the ability of AAV-13A7-Fc and AAV-14D5-dimHLE to modulate inflammation in vivo, we used a model of acute colitis induced by DSS. This in vivo experimental system in rodents recapitulates multiple aspects of human IBD at clinical, microscopic and molecular levels [35]. Mice received a single i.m. injection of AAVnano coding for nanobody-based biologics, and three weeks later they were given 2.5% DSS in drinking water for five days followed by eight days of regular water. We observed that the disease activity index (DAI) progressively increased after DSS administration in both untreated and treated mice up to day 7, with no difference among groups (Fig. 5A). Remarkably, after this time point the DAI of AAV-13A7-Fc treated mice remained significantly lower compared to phosphate-buffered saline (PBS)- or AAV-14D5-dimHLE-mice. Mice injected with AAV-14D5-dimHLE exhibited a trend toward a worsening of disease as compared to untreated mice that did not reach statistical significance, possibly reflecting an already very high level of inflammation. Comparing the three groups of mice, the cumulative DAI score from day 8 was 10.85 ± 2.29 in AAV-13A7-Fc treated mice vs 16.75 ± 3.02 in control mice and 18.90 ± 2.40 in AAV-14D5-dimHLE treated mice. In correlation with clinical data, a reduction of colon length and signs of inflammation (redness and swelling) were observed macroscopically in PBS-treated mice, which were ameliorated by AAV-13A7-Fc treatment and slightly worsened by AAV-14D5-dimHLE (Fig. 5B). At microscopic level, colons from DSS-treated mice exhibited mucosal and submucosal immune cell infiltration and focal crypt damage with goblet cell loss (Fig. 5C). These histopathological features of intestinal inflammation were significantly reduced in AAV-13A7-Fc-injected mice. Indeed, the average histopathological score was 3.75 ± 0.79 for PBS-treated mice vs 1.63 ± 0.32 for AAV-13A7-Fc-treated mice (Fig. 5C). AAV-14D5-dimHLE treatment did not significantly worsen this score, with nonetheless a trend for a higher score in untreated mice (4.4 ± 1.11). Notably, protective effects of AAV-13A7-Fc on clinical and histopathological scores were observed even when a higher dose of DSS (3%) was used, suggesting that even higher levels of inflammation could be treated using biologics that block the P2X7 pathway (Additional file 1: Fig. S6).

**Fig. 6:**
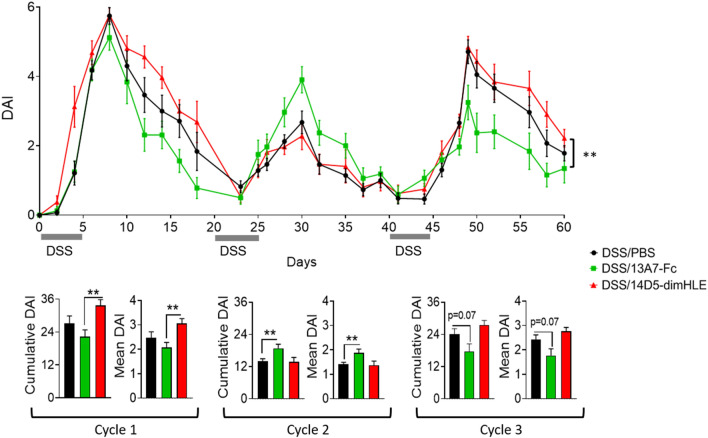
AAV-13A7-Fc administration ameliorated chronic DSS-induced colitis. Mice (n = 10) received 10^11^ vg of AAV coding for 13A7-Fc or 14D5-dimHLE and received three cycles of 2.5% DSS in drinking water for 5 days followed by regular water for 15 additional days. Disease activity score (DAI) curves and cumulative and mean DAI scores are shown. Mean ± SEM are shown. Statistical comparison two-way ANOVA (top) or one-way ANOVA (bottom) were performed with *p < 0.05 and **p < 0.01 and ***p < 0.001. A representative experiment out of three is shown

In addition to a model of acute colitis induced by DSS, we used a chronic model based on the administration of several cycles of DSS/water (*i.e*., here three cycles of 2.5% DSS given for five days, followed by 15 days of regular water), and studied the effect of a single AAVnano injection on the clinical development of the disease (Fig. 6). As above, we observed a significant effect on the average clinical score during the first cycle of DSS-treatment, with a reduction in the case of 13A7-Fc and an increase for 14D5-dimHLE. The second cycle of DSS-treatment was less informative and only induced a 1.5- to twofold increase in clinical scores, possibly because inflammation induced by the first DSS treatment was not yet resorbed at the end of the first cycle. Nevertheless, the responses to the third cycle of DSS were of a higher amplitude and we observed that the disease reduction induced by AAV-13A7-Fc and the increase by AAV-14D5-dimHLE treatments persisted (Fig. 6). This suggests that targeting P2X7 by nanobody-based biologics leads to efficient P2X7 modulation even in a context of chronic inflammation.

### AAV-13A7-Fc protects from CD62L and CD27 shedding in vivo even after DSS administration

**Fig. 7:**
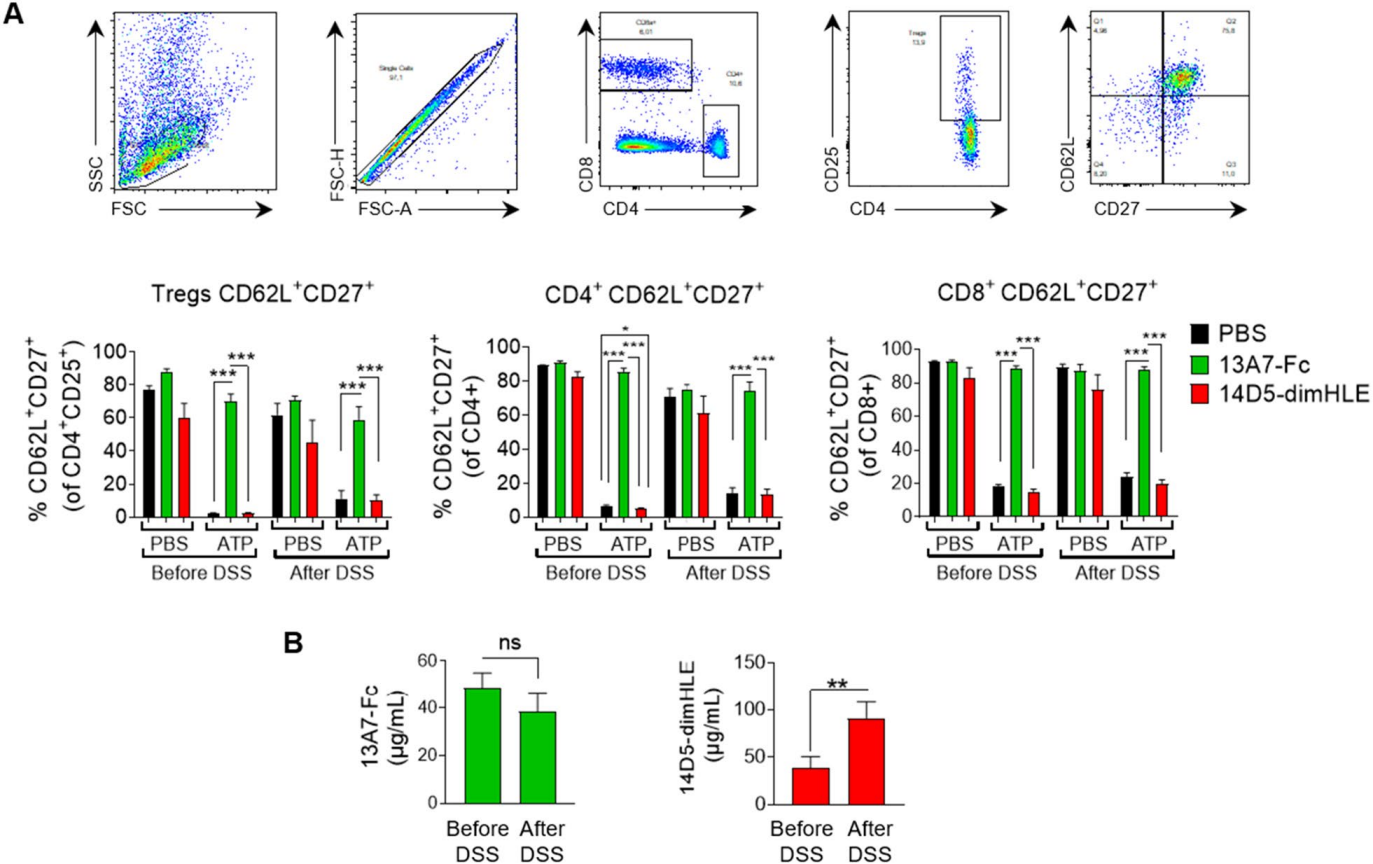
Blocking effect of AAV-13A7-Fc administration on CD27 and CD62L shedding during DSS administration.** A** Blood samples were collected three weeks after AAV-13A7-Fc or AAV-14D5-dimHLE i.m. injection (before DSS) or 13 days later (*i.e*., after DSS 2.5% for 5 days followed by 8 days of regular water). CD62L and CD27 shedding was evaluated by flow cytometry of the indicated gated T cell subsets after incubation, or not, with 150 µM ATP. The top panels illustrate the flow cytometry gating strategy and the bottom panels the percentage of CD27^+^CD62L^high^ in each indicated T cell subset. **B** Plasma samples were collected before the start of DSS treatment (three weeks after AAV injection) and after 2.5% DSS + regular water treatment (day 13). Concentrations of unbound nanobody-based biologics were titrated by flow cytometry using a P2X7-expressing cell line and standard curves obtained with the same recombinant biologics produced in vitro. One representative experiment out of three is shown, with n = 5–6 mice per group in each experiment. Statistical analysis was performed using one-way ANOVA with *p < 0.05, **p < 0.01 and ***p < 0.001

As a readout of the ability of our AAVnano methodology to modulate P2X7 activity before and after disease induction, we evaluated the shedding of CD27 and CD62L in peripheral blood lymphocytes from AAV-13A7-Fc and AAV-14D5-dimHLE injected mice, induced by exposure to 150 µM ATP ex vivo. In PBS-injected mice, ATP treatment dramatically reduced the percentage of CD62L^+^CD27^+^ cells within CD4^+^, CD8^+^ and Treg (CD4^+^CD25^+^) populations compared to cells not exposed to ATP (Fig. 7A). As expected, lymphocytes from AAV-13A7-Fc-injected mice were protected from ATP-induced shedding of CD27 and CD62L, in agreement with our previous experiments. Importantly, this protective effect was maintained after DSS-treatment suggesting that the in vivo production of 13A7-Fc biologics was not affected by the intense inflammatory status induced by DSS administration (Fig. 7A). To further validate this point, we evaluated the concentration of 13A7-Fc biologics in sera collected from DSS-treated animals. Data confirmed that the concentrations of unbound 13A7-Fc found in the circulation were maintained above 30 µg/ml and were not significantly affected by DSS-treatment (Fig. 7B). Similar to the situation concerning the functional effect and the concentration of 14D5-dimHLE biologics, our data confirmed a slightly potentiating effect of this biologic in the experimental condition used, and an even higher production of this biologic in the circulation over time, even after DSS treatment (Fig. 7A, B).

### AAV-13A7-Fc reduces chemokine and proinflammatory cytokine expression in colons from DSS-treated mice

**Fig. 8:**
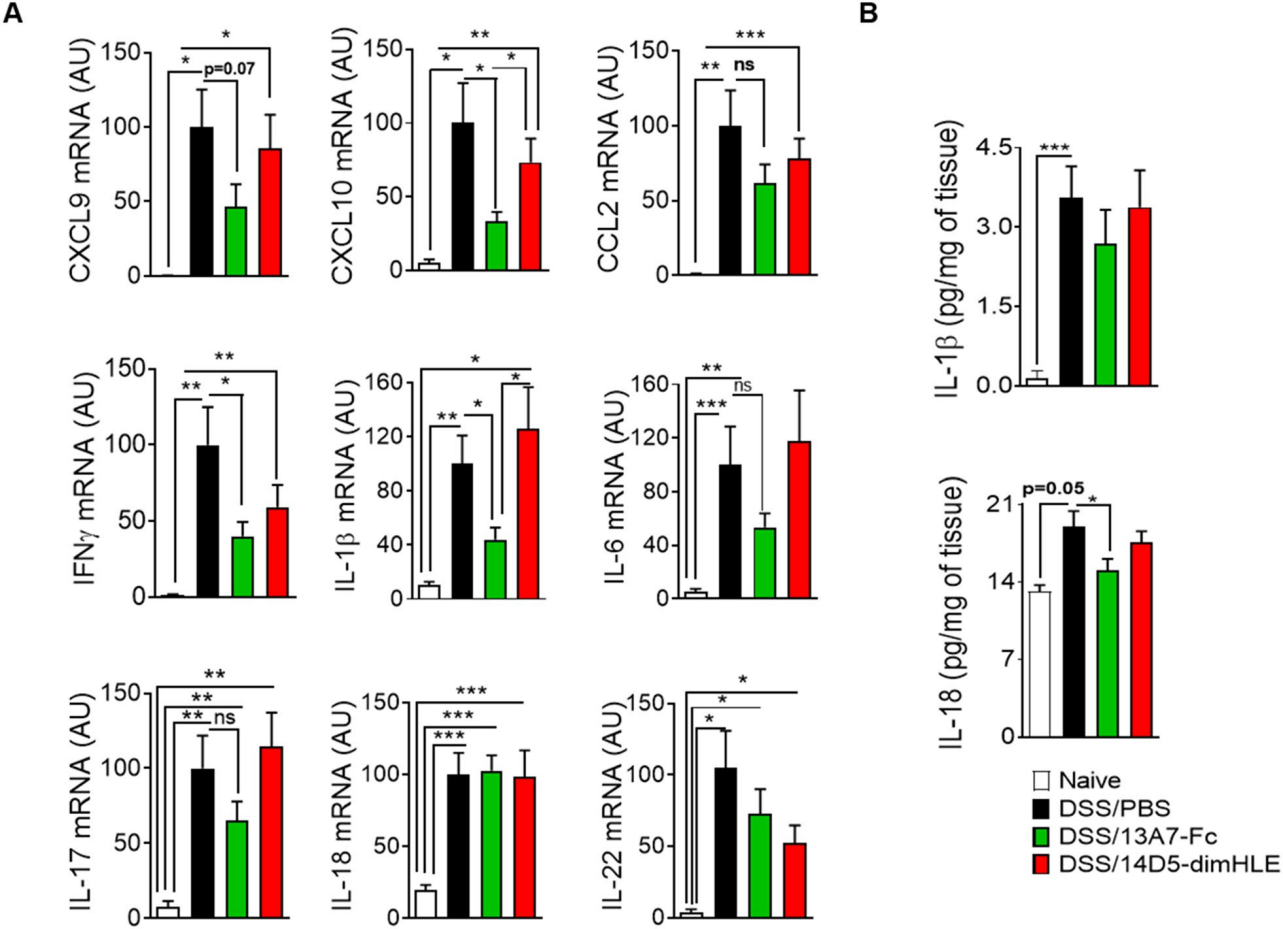
Chemokine and proinflammatory cytokine production was reduced in mice receiving AAV-13A7-Fc. Mice (n = 10) receiving AAVnano coding for 13A7-Fc or 14D5-dimHLE at a dose of 10^11^ vg/mouse, were given, three weeks later, 2.5% DSS in drinking water for five days. Colons were collected on day 13 for chemokine and cytokine assessment. **A** mRNA expression of multiple chemokines (CXCL9, CXC10 and CCL2) and cytokines (IFNγ, IL-1β, IL-6, IL-17, IL-18 and IL-22) in the colons of mice injected with AAV coding for 13A7-Fc or 14D5-dimHLE and exposed to DSS (day 13 from the beginning of DSS treatment). **B** IL-1 β and IL-18 levels in the supernatants of colon explants after 16 h of culture. Results represent the combined data of two different experiments normalized to the DSS-treated group expression values. Statistical analysis was performed by one-way ANOVA with *p < 0.05, **p < 0.01 and ***p < 0.001

DSS induces intestinal inflammation through the disruption of the intestinal epithelium, enabling the entry of luminal bacteria and associated antigens into the mucosa leading to an inflammatory response [35]. Immune cells, recruited by means of locally produced chemokines, produce a wide range of proinflammatory mediators amplifying the immune response and leading to tissue damage. Indeed, expression of the chemokines CXCL9, CXCL10 and CCL2 and the proinflammatory cytokines IFNγ, IL-1β, IL-6, IL-17, IL-18 and IL-22 in colon tissue was significantly elevated in DSS-treated mice compared to untreated mice (Fig. 8A). In agreement with our clinical data and histological analysis, i.m. administration of AAV-13A7-Fc reduced the mRNA expression of all these chemokines and cytokines except for IL-18. AAV-14D5-dimHLE-treated mice had a trend to express higher levels of IL-1β, IL-6 and IL-17 in line with our initial hypothesis, but also displayed a trend to a reduced expression of all other molecules.

P2X7 promotes inflammation by activating the NLRP3-inflammasome, and participates thereby not only in the maturation of IL-1β and IL-18 that occurs in the cytoplasm, but also in their release to the extracellular compartment. Therefore, we also evaluated the levels of these cytokines, at the protein level, in culture supernatants of colon explants. In agreement with P2X7 inhibition and amelioration of the disease, we observed a reduction in the level of these cytokines in AAV-13A7-Fc injected mice as compared to the PBS control group that reached statistical significance for IL-18 (Fig. 8B).

## Discussion

Recombinant AAV vectors represent an efficient means to produce therapeutic proteins in vivo, both in animal models as well as in humans [36–40]. Transgenes transferred using AAV vectors persist for months in episomal form (*i.e.* unintegrated extrachromosomal form), considerably limiting the risk of mutational insertion as compared to other types of vectors. Additionally, their lack of pathogenicity, their physical stability and their ability to infect non-dividing cells, render AAV vectors ideal for efficient gene transfer in animal models and in the clinic. Compared to other viral vectors, AAV vectors are less immunogenic than adenovirus vectors, are as easily produced and titrated as lentivirus vectors and—as non-pathogenic and non-replicative dependoviruses—are safer to manipulate than adenovirus or lentivirus vectors. Additionally, a variety of capsid serotypes offer the possibility to selectively target tissue of interest [41]. For instance, AAV1 displays a high transduction efficiency and tissue selectivity for skeletal muscle, AAV8 for muscle and liver, and AAV9 for brain. Given their good safety profile, AAV vectors have been widely used in the past decades with 255 clinical trials registered by June of 2022 and six AAV products receiving approval from the FDA or EMA in recent years: for the treatment of lipoprotein lipase deficiency (AAV1, Glybera), retinal dystrophy (AAV2, Luxturna), spinal muscular atrophy (AAV9, Zolgensma), haemophilia B (AAV5, Hemgenix), Duchenne muscular dystrophy (AAVrh74, Elevidys) and, haemophilia A (AAV5, Roctavian) [42, 43].

It was previously shown that AAV-mediated gene transfer in muscle can be used to induce the systemic secretion of broadly-neutralizing antibodies against human immunodeficiency virus (HIV), thereby protecting humanized-mice against HIV infection [44, 45]. This approach, named “vectored immunoprophylaxis” (VIP), induced the appearance and persistence (for months) of anti-HIV antibodies in the circulation of mice that had received a single i.m. injection of AAV1 vector coding for the selected antibody. A second study confirmed the efficiency and feasibility of the VIP strategy to confer durable protection also against influenza infection in mice [44]. Here, we adapted this methodology to induce long-term expression of nanobody-based biologics, selected for their remarkable ability to either fully block (13A7-Fc), or to potentiate (14D5-dimHLE), P2X7 functions in vivo, and evaluated their effects in a DSS-induced inflammatory colitis animal model. We demonstrated that AAV1-mediated gene transfer in muscle cells, following a single i.m. injection of a given AAVnano vector, is sufficient to drive effective expression of nanobody-based biologics in vivo and to modulate P2X7 functions in a time frame ranging from 2 weeks to at least 4 months post AAV-injection. Also, nanobody production by transduced muscle cells in vivo was remarkably not affected by intestinal inflammation, and the effect persisted after DSS-induced colitis (Fig. 7). We suggest that this AAVnano methodology is not only valuable for validating the importance of P2X7 as a target in a variety of pathophysiological contexts, but also for screening different formats of nanobody-based biologics for clinical development. Interestingly, a recently described humanized P2X7 receptor knock-in mouse model was generated [46], which may be useful for such a screening strategy using available functional nanobodies against human P2X7 [28].

Unexpectedly, we found that the format of the nanobody-based biologic can influence its biological activity when used in vivo as illustrated in our study when comparing the biological effects of 14D5-dimHLE and 14D5-Fc. While, 14D5-dimHLE behaves as a potentiating allosteric modulator of P2X7, as expected from the in vitro data, 14D5-Fc was unexpectedly found to behave as a weak antagonist of P2X7. This could possibly be related to steric hindrance, or more likely, to the induction of P2X7-endocytosis. This hypothesis remains however to be studied and may possibly depend on concomitant binding to FcRs on the target cells. Regardless of the precise mechanism, this has highlighted the need to validate candidates for nanobody based biologics not only in vitro, but also in vivo and in kinetic studies as we did in the present study, before using them in prophylactic or therapeutic approaches in relevant pathophysiological animal models.

Whereas healthy tissue contains minimal levels of extracellular nucleotides and nucleosides, their levels increase in certain conditions such as hypoxia, ischemia, inflammation, or cancer [47]. In line, it has been reported that ATP is released in colon tissue during DSS-induced colitis in mice [48]. Indeed, it was shown that the levels of eATP progressively increased from day 3 of treatment with 4% DSS, with the highest levels at day 7. Interestingly, we started to observe a protection from DSS-induced disease at a clinical level by AAV-13A7-Fc only from day 7 onwards, which correlates with the period when the highest concentrations of eATP were detected. In the same study, Wan et al. demonstrated that the blockade of ATP release by carbenoxolone (CBX), or promoting ATP degradation by a potent ATPase (apyrase), reduced eATP levels in the colon and attenuated DSS-induced colitis [48]. Conversely, tissue damage in mice with colitis was exacerbated by the inhibition of eATP degradation by sodium metatungstate (POM-1, a known inhibitor of ecto-nucleotidases). This supports the hypothesized role of eATP in the pathogenesis of DSS-induced colitis in mice, and suggests a role for P2X7. In a different study, the potential therapeutic effect of P2X7 blockade in rat DSS-induced colitis was evaluated using Brilliant Blue G (BBG), a widely used non-peptidic P2X7 antagonist [23]. BBG ameliorated colitis induced by DSS, restoring colonic macroscopic and microscopic morphology. This effect was accompanied by reduced expression of MyD88, NF-κB, IL-6, TNF-α and P2X7. In addition, BBG reduced NLRP3 inflammasome activation, subsequent caspase-1 activation and IL-1β and IL-18 release. The combined administration of OLT1177, an NLRP3 inhibitor, with BBG potentiated its inhibitory effect on NLRP3, caspase-1, IL-1β and IL-18 levels. Using another model of colitis, other authors reported that prophylactic intraperitoneal administration of BBG significantly reduced inflammatory response in the colon of rats treated with TNBS [49]. In a different study with a prophylactic/curative approach, administration of BBG starting one hour after TNBS-colitis induction in rats led to an amelioration of clinical and histopathological signs of the disease [50]. Although these are interesting results suggesting the involvement of P2X7 in the pathogenesis of inflammatory colitis, the specificity of BBG has been questioned, as data have shown that BBG can also antagonize P2X1, P2X2, P2X3, and P2X4 receptors to some extent, as well as voltage-gated sodium channels [51]. In addition, compared to our approach, where a single injection of AAV-13A7-Fc was sufficient to obtain high concentrations of nanobody-based biologics in the serum and significant therapeutic effects, BBG was administered either every day [23, 50], or before and three days after colitis induction [49]. A recent study also reported the development of novel pyroglutamide-based P2X7 antagonists, with two compounds exhibiting beneficial effects in a DSS-induced colitis model. Although these are all promising results, small drugs were administered on a daily basis [52], whereas nanobody-based biologics represent an alternative single therapeutic treatment that offers target specificity as well as longer half-life in vivo.

Our data demonstrates that AAVnano methodology (*i.e*., AAV coding for nanobody-based biologics) could be used to either inhibit or potentiate P2X7 activity in vivo. Their i.m. administration modulates inflammation in vivo, consequently leading to an amelioration or an increase of DSS-induced colitis, respectively. This modulation could be observed three weeks after AAVnano injection in acute inflammatory conditions induced by one cycle of DSS treatment (Fig. 5). Moreover, consistent with an efficient long-term modulation of P2X7 in vivo, our results obtained after one cycle of DSS were recapitulated in a chronic model of colitis after three cycles of DSS (Fig. 6). Interestingly, these effects were observed during the third but not the second cycle of DSS. Although it remains to be explored, this might be explained by differences in the status of the mice before the administration of DSS in each cycle (i.e., non-exposed vs. exposed to one or two cycles of DSS). A milder disease to a second DSS exposure has also been reported in other studies [53–55]. Also, this difference of sensitivity to the first and second cycles of DSS may be related to the kinetics of epithelial mucosa healing after the first DSS exposure. Indeed, in the AAV-13A7-Fc group, healing after the first cycle appeared faster and we observed a slightly higher clinical response to the second DSS cycle as compared to the other groups that however remained of lower intensity compared to the amplitudes reached after the first and third DSS treatments. Interestingly, although not specifically addressed in the present study, P2X7 itself may also be implicated in the healing phase where epithelial cell proliferation contributes to the reestablishment of the intestinal barrier. In a previous study, we observed that P2X7 blockade enhanced proliferation of intestinal epithelial cells and protected them from apoptosis [24]. The accelerated recovery in AAV-13A7-Fc-treated mice might have reconstituted intestinal integrity faster than that in untreated mice. In such a scenario, P2X7 blockade in a context of colon inflammation may not only contribute to a reduction of inflammasome activation and production of pro-inflammatory cytokines by immune cells, but may also foster a better restoration of the epithelial barrier, thereby limiting perpetuation of the inflammatory process.

In the present study we did not investigate the curative effect of nanobody-based biologics and we did not study either the precise cellular targets of our protective nanobody-based biologics that remain to be evaluated in future studies. As different immune and non-immune cells express P2X7, this question remains to be fully addressed using for instance conditional P2X7 knock-out mice that are under development. However, given that myeloid cells express P2X7 and have been implicated in the pathogenesis of inflammatory colitis, this cell subset may be involved in the protective effects that we observed in the present study. Indeed, P2X7-dependent and P2X7-independent activation of NLRP3-inflammasome assembly in myeloid cells, are known to represent important sources of proinflammatory cytokines such as IL-1β and IL-18, whose production was reduced in colon explants from AAV-13A7-treated mice (Fig. 8). Further supporting their role, a recent study demonstrated that targeting estrogen receptor β diminished inflammatory lesions in TNBS-induced colitis in rats, via down-regulating P2X7 expression in macrophages [56]. Moreover, further mechanistic information may be obtained by using other models of the disease such as a TNBS model, where anti-hapten T cells represent the main drivers in the initial steps of the disease. Given the potential implication of P2X7-expressing T cells in the disease as well as their role in mucosal adaptive immunity [11, 21], T-cell transfer models of colitis may be used in future studies to evaluate their role using the AAVnano methodological approach that was used here, or using direct injections of small-molecule or biologic inhibitors of P2X7.

In our study we focused on a novel class of therapeutic agent based on nanobody-based biologics. Compared to small-molecules, biologics may improve target-specificity, and avoid some of the drawbacks [57]. In our study, we did not observe noticeable side effects of the nanobody-based biologics evaluated, despite their continuous production in vivo for more than 100 days using our AAV-based gene transfer approach. Hence, our data are compatible with a remarkable efficacy/safety profile that paves the way for future development of nanobody-based therapeutics.

## Conclusions

In summary, our data demonstrate that nanobody-based biologics can be produced in vivo following a single i.m. administration of an AAV vector in vivo for several months without conspicuous side effects. Using this strategy to express nanobody-based biologics targeting P2X7, we have demonstrated beneficial effects of a P2X7-blocking biologic on DSS-induced colitis, with a reduction of clinical symptoms, colon histopathology as well as reduced expression of chemokines and proinflammatory cytokines. Our results highlight the beneficial role of P2X7 blockade in both acute and chronic inflammatory conditions, which may apply also to other disease models. Moreover, the P2X7-blocking/potentiating strategy explored here represents a valuable novel approach for assessing the role of P2X7 in a variety of inflammatory diseases as well as other pathophysiological contexts in which P2X7 is suspected to play an important role, ranging from cancer, to autoimmune and neurodegenerative diseases. In a context of intestinal inflammation, our data validate the beneficial role of P2X7-inhibition and suggest that nanobody-based biologics represent a novel class of pharmacological drugs with high potency and selectivity that may complement the already available therapeutic strategies.

## Methods

### Animal experiments

Eight-week-old C57BL/6JRj female mice (Janvier Labs) were used in these studies. Mice were maintained in an SPF-free vivarium at conditions of constant temperature (21 ± 2 °C) under a controlled 12-h/12-h light–dark cycle (lights on at 7:00 A.M.). Mice were housed in groups of five mice per cage with ad libitum access to food and water. Environmental enrichment was provided with cardboard houses and compressed cotton nesting squares. All efforts were made to minimize animal suffering and to reduce the number of animals. Animals were checked daily for signs of suffering and were euthanized if weight loss was > 20%, or if hunching posture, shivering, lack of movement or cold temperature were observed. Wet food pellets were placed on the bed cage when the animals began to develop clinical signs to facilitate access to food and hydration, and injections of physiological serum were given when necessary to mice exhibiting signs of severe dehydration (i.e. sunken eyes and fuzzy fur). All mice were treated with acetaminophen (0.4 mg/ml in drinking water) from day 6 after dextran sodium sulfate (DSS) administration, which corresponds to the appearance of the first clinical signs. Mice were sacrificed by cervical dislocation. For all experiments, mice were randomly assigned to the different groups of treatment. For DSS experiments, each cage contained mice from each treatment group (PBS, 13A7-Fc, and 14D5-dimHLE) in order to avoid confounding effects due to variability among cages. For our DSS experiments, a group size of 10 mice (n = 10) was selected based on our previous experience [58], to ensure a statistical significance considering the biological variability of disease development and possible loss of mice due to over 20% weight loss.

### Generation of AAV vectors coding for nanobody-based biologics

Detailed information regarding the nanobodies and the AAV-delivery system used in our laboratory has been previously published [31, 59]. The construct 14D5-dimHLE used in this study was based on a nanobody dimer (“dim” format) fused to the Alb8 anti-albumin nanobody (half-life extended “HLE” format). For that, the coding sequences corresponding to two 14D5 nanobodies were fused using a 35-GS linker (GGGGS) × 7. This construct was further fused to the anti-albumin nanobody Alb8 via a 9-GS linker (GGGGSGGGS) [28, 60]. The P2X7-blocking 13A7-Fc was constructed by fusing the sequence corresponding to the nanobody 13A7 to the hinge and Fc regions of a mutated mouse IgG1 antibody carrying the “LSF” mutations (T252L, T254S, T256F) [61] described previously to confer a higher affinity to the neonatal Fc receptor (FcRn) and thereby an extended half-life in vivo.

For the production of AAV, all constructs were cloned into a pFB plasmid under the control of a CBA promoter (for AAV1 constructs coding for 14D5-dimHLE or 13A7-Fc). Production, purification, and titration of AAV1 were performed by Virovek (Hayward, California, USA) using the baculovirus expression system in Sf9 insect cells. For muscle transduction, mice hind legs were shaved under ketamine/xylazine anesthesia and 100 µL diluted AAV were injected into four gastrocnemius or quadriceps muscle sites (25  µl per injection site) to reach a total dose of 10^11^ viral genomes (vg) per mouse.

### Evaluation of the blocking or potentiating capacity of the nanobody-based biologics produced in vivo

Evaluation of the agonistic or antagonistic potential of each nanobody was determined ex vivo using a functional assay based on ATP-induced and P2X7-dependent shedding of CD62L or/and CD27 at the surface of lymphocytes [8]. Briefly, blood samples were collected from the retroorbital sinus in EDTA-containing tubes, and peripheral blood mononuclear cells were treated with or without ATP (at doses ranging from 30 to 600 μM, depending on the study) in PBS for 15 min at 37 °C. For some studies, similar experiments were performed with splenocytes obtained at the study endpoint after collection and preparation of splenocytes and filtration through a 40 μm cell-strainer. After incubation with ATP, cells were washed with PBS containing 3% FBS and stained with a cocktail of directly labelled antibodies containing anti- CD4-APC-Cy7, CD25-PE, CD8a-PE-Cy7, CD62L-FITC, CD27-APC. Cells were incubated with antibodies for 30 min at 4 °C, washed with PBS containing 3% FBS. Erythrocyte lysis and cell fixation were performed simultaneously by incubation with RBC Lysis/Fixation Solution (Sony Biotechnology) for 30 min at room temperature, before washing and analysis by flow cytometry using an LSRFortessa (BD Biosciences).

### Titration of the nanobody-based biologics produced in vivo

Nanobody levels in serum were assessed by flow cytometry as follows. B16F10 cells transduced to stably express P2X7 were used as a target cell in vitro (B16F10-P2X7). Cells were incubated with TruStain FcX™ (anti-mouse CD16/32) (Sony Biotechnology) for 15 min at 4 °C, followed by 30 min of further incubation with 15 μl of diluted serum collected from mice injected with the AAV vector coding for the selected nanobody-based biologic. After washing with PBS containing 3% FBS, 13A7-Fc biologics bound at the surface of B16F10-P2X7 cells were detected using a biotinylated antibody directed against the Fc moiety (anti-mouse IgG1, Sony Biotechnology) for 30 min at 4 °C. Cells were then washed and incubated with streptavidin-PE (Sony Biotechnology) for 30 min at 4 °C. For detection of 14D5-dimHLE at the surface of B16F10-P2X7 cells, an antibody directed against the anti-albumin nanobody was used (mAb77). Cells were analyzed with an LSRFortessa (BD Biosciences) flow cytometer. Calculation of the concentration of each nanobody-based construct contained in serum was performed by comparison with a standard curve obtained after serial dilution of the same recombinant construct obtained and purified from transiently transfected HEK-293 cells.

### Colitis induction following administration of dextran sodium sulfate

Mice (n = 10/group) received an i.m. injection of either PBS, 10^11^ vg of AAV-13A7-Fc, or 10^11^ vg of AAV-14D5-dimHLE. Mice were randomized for treatment. Three weeks later, acute inflammation was induced by administration of 2.5% DSS in drinking water from days 1 to 5, after which they were switched to regular water. For the chronic DSS model, mice were given three cycles of 2.5% DSS for five days followed by 15 days of water. The disease activity index (DAI), was evaluated on a daily basis by two independent researchers blinded to the treatment, following a scoring system from 0 to 10, as reported previously [24]. DAI was calculated by the addition of three different clinical scores: (1) body weight loss, which was scored from 0 to 4 (0, no loss; 1, loss < 5%; 2, loss < 10%; 3, loss < 20%; 4, loss > 20%); (2) stool consistency, from 0 to 3 (0, normal; 1, loose stools; 2, watery diarrhea; 3, severe watery diarrhea); (3) presence of rectal bleeding, from 0 to 3 (0, no blood; 1, presence of petechia; 2, traces of blood in the stools; 3, bleeding). Mice were sacrificed on day 13, and colons, mesenteric lymph nodes and spleens were collected for further mechanistic studies. Colons were washed and divided in three portions as follows: starting from the proximal colon, 0.5 cm of tissue was collected for RNA studies, the next 0.5 cm for explant cultures, and the rest was rolled using the “Swiss Roll” technique and fixed overnight in 4% paraformaldehyde for histological studies.

### Histopathological evaluation

Tissues were transferred to 70% ethanol and progressively dehydrated for paraffin embedding. Seven-μm sections were stained with hematoxylin and eosin. Histological inflammation was scored in a blinded fashion from 0 to 10 according to four cumulative parameters: (1) severity of inflammation, from 0 to 3 (0, none; 1, mild; 2, moderate; 3, severe), (2) extent of inflammation from 0 to 3 (0, none; 1, mucosa; 2, mucosa and submucosa; 3, transmural), (3) crypt damage from 0 to 3 (0, none; 1, basal to 1/3; 2, basal to 2/3; 3, crypt loss, crypts, and epithelium loss), and percentage of tissue affected by inflammation from 0 to 4 (0, 0%; 1, 25%; 2, 50%; 3, 75%; 4, 100%).

### Real time RT-PCR

RNA was isolated from colons with Trizol reagent (Sigma) and retrotranscribed with the ImProm-II™ transcription system from Promega. Real time quantitative PCRs were performed using the Lightcycler 480 SYBR Green I Master (Roche). Primers are listed in Additional file 1: Table S1. Amplification was performed with a LightCycler 480 System SW 1.5.1 (Roche) as follows: initial denaturation at 95 °C for 10 min, 40 cycles of 95 °C for 30 s followed by 60 °C (for CCL2, CXCL9, IFNγ, IL-1β, IL-6, IL-17, IL-18, IL-22 and TGFβ) or 62 °C (for CXCL10) for 30 s, and 72 °C for 30 s. Melting curve analysis confirmed primer specificity. The calculation was normalized to the housekeeping gene Eef2 according to the formula (E_target_)Δ^Ct^_target_/(E_normalizer_)Δ^Ct^_normalizer_. Data were normalized with respect to DSS PBS-injected controls (100% expression) in order to correct for variation between experiments.

### Colon explant cultures

Colon samples were excised longitudinally, rinsed in PBS and incubated in 0.8 ml of RPMI 1640 medium with 10% FBS and 1% penicillin and streptomycin in 24-well plates for 16 h. Supernatants were collected and stored at − 20 °C for cytokine evaluation by ELISA. Cytokine levels were normalized with respect to the weight of the explant in culture (mg) in order to correct for difference in tissue size.

### Data and statistical analyses

The data and statistical analyses comply with the recommendations on experimental design and analysis in pharmacology [62]. Data are presented as mean ± SEM. Two-Way ANOVA with Dunnett’s multiple comparisons post hoc tests was used for statistical analysis of DAI curves. Comparisons between groups were performed using one-way ANOVA and Tukey’s multiple comparisons post hoc tests. For all, the threshold for statistical significance was set at p < 0.05 (*p < 0.05, **p < 0.01 and ***p < 0.001). Statistical analyses were performed using GraphPad Prism 8.0 software (GraphPad Software Inc., USA).

### Supplementary Information

**Additional file 1:** **Figure S1.** Evaluation of the blocking effect of AAV-13A7-Fc vector on the activity of P2X7 on the surface of CD4^+^ T cells 12 weeks after i.m. administration of different doses of AAVnano vector. AAV-13A7-Fc vector was injected i.m. with the indicated AAVnano dose and blood cells were collected 12 weeks later to evaluate the functional activity of P2X7 on the surface of CD4^+^ T cells after incubation with the indicated dose of ATP ranging from 150 µM to 600 µM ATP. The flow cytometry profiles illustrate the P2X7-dependent shedding of CD62L from the gated CD4^+^ T cells after incubation with the indicated dose of ATP in mice injected 12 weeks earlier with the indicated dose of AAV-13A7-Fc vector. The indicated numbers correspond to the percentages of CD62L^high^ cells in each experimental condition after treatment with ATP. The graph bars recapitulate the data obtained in this experiment for each indicated dose of AAV-13A7-Fc vector. **Figure S2.** Evaluation of the blocking effect of AAV-13A7-Fc vector on the activity of P2X7 on the surface of CD8^+^ T cells 12 weeks after i.m. administration of different doses of AAVnano vector. AAV-13A7-Fc vector was injected i.m. with the indicated AAVnano dose and blood cells were collected 12 weeks later to evaluate the functional activity of P2X7 on the surface of CD8^+^ T cells after incubation with the indicated concentration of ATP ranging from 150 µM to 600 µM. The flow cytometry profiles illustrate the P2X7-dependent shedding of CD62L from the gated CD8^+^ T cells after incubation with the indicated dose of ATP in mice injected 12 weeks earlier with the indicated dose of AAV-13A7-Fc vector. The indicated numbers correspond to the percentages of CD62L^high^ cells in each experimental condition after treatment with ATP. The graph bars recapitulate the data obtained in this experiment for each indicated dose of AAV-13A7-Fc vector. **Figure S3.** Evaluation of AAVnano toxicity. Mice (n = 5) received i.m. 10^11^ vg of AAV coding for RFP (AAV control), 13A7-Fc or 14D5-dimHLE. **A** Weight follow up over time (% of original weight). **B** Serum enzymatic activity of alanine amino transferase (ALT) and creatine kinase (CK) measured on day 30 post-injection. We used the concanavalin A (ConA) liver toxicity model as a positive control. For that, 5 mice were injected i.v. with ConA (20 mg/kg), and serum was collected 8 h later. **C** Representative images of muscle, liver, kidney and colon sections from AAV-injected mice stained with hematoxylin and eosin (scale bar correspond to 200 µm; n = 5 mice/group). Representative images of control mice (naïve or PBS injected, n = 5 mice/group) are shown on the right panels. **Figure S4.** Evaluation of the AAV-13A7-Fc blocking and of the AAV-14D5-dimHLE potentiating vectors on the activity of P2X7 overtime upon a single i.m. injection of each AAVnano vector. As in Fig. 4, AAV-13A7-Fc or AAV-14D5-dimHLE vectors were injected i.m. at a dose of 10^11^ vg/mouse and blood cells were collected at the indicated time points. Blood cells were incubated with 150 µM or 30 µM ATP, as indicated, and the percentages of CD4^+^CD62L^high^ cells were determined by flow cytometry as an evaluation of P2X7 activity on the surface of the gated CD4^+^ T cells. The first two bars in each graph correspond, respectively, to negative and positive control cells collected from control mice receiving AAV-RFP control AAV vector, treated or not with the indicated concentration of ATP. Results represent mean values ± SEM, with n = 5 mice per group. **Figure S5.** Comparison of P2X7 activity on the surface of T cells collected from AAV-13A7-Fc injected mice or from P2X7^−/−^ mice. AAV-13A7-Fc vector was injected i.m. at a dose of 10^11^ vg/mouse and splenocytes were collected 120 days later to evaluate the functional activity of P2X7 on the surface of the indicated T cell subset and were compared to T cells collected from P2X7^−/−^ mice using the same experimental conditions. **A**. Histogram overlay showing expression of CD27 on the surface of CD4 + cells collected from untreated P2X7-deficient mice (green histograms) or from the WT mice that were treated as indicated (red histograms). Bar graphs showing the percentage of CD27^+^ cells (**B**), or CD27^+^CD62L^high^ (**C**) in the indicated gated T cell subset. **Figure S6.** AAV-13A7-Fc administration ameliorated high-dose DSS-induced colitis. Mice (n = 10) received 10^11^ vg/mouse of AAV coding for 13A7-Fc or 14D5-dimHLE and three weeks later they were given 3% DSS in drinking water. Mice were switched to regular water for eight additional days and colons were collected at the end of the study. **A**. Disease activity score (DAI) from 13A7-Fc and PBS-injected mice with a higher dose of DSS (3%). **B**. Colon macroscopic appearance of naïve, untransduced and 13A7-Fc mice. **C**. Histopathological analysis of colon stained with hematoxylin and eosin staining. **D**. mRNA expression of chemokine CXCL10 and cytokines (TGF-β, IL-1 β, IL-6, IL-18 and IL-22) in the colons of mice injected with AAV coding for 13A7-Fc and exposed to DSS (day 13 from the beginning of DSS treatment). Mean ± SEM are shown. For statistical comparison two-way ANOVA (**A**) or one-way ANOVA (**B** and **C**) were performed with *p < 0.05 and **p < 0.01. **Table S1.** List of RT-qPCR primers.

## Data Availability

The data that support the findings of this study are available from the corresponding author upon reasonable request.

## Abbreviations

AAV: Adeno-associated virus
eATP: Extracellular ATP
DSS: Dextran sodium sulfate
HLE: Half-life extended
IBD: Inflammatory bowel disease
i.m.: Intramuscular
Nb: Nanobodies
Treg: Regulatory T cells
