# Supplementary material for: Administration of an AAV vector coding for a P2X7-blocking nanobody-based biologic ameliorates colitis in mice
Source: J Nanobiotechnology. 2024 Jan 11;22:27. doi: 10.1186/s12951-023-02285-4 (PMC10785547; doi:10.1186/s12951-023-02285-4)
Supplement: Supplementary file 1 — Additional file 1: Figure S1. Evaluation of the blocking effect of AAV-13A7-Fc vector on the activity of P2X7 on the surface of CD4+ T cells 12 weeks after i.m. administration of different doses of AAVnano vector. AAV-13A7-Fc vector was injected i.m. with the indicated AAVnano dose and blood cells were collected 12 weeks later to evaluate the functional activity of P2X7 on the surface of CD4+ T cells after incubation with the indicated dose of ATP ranging from 150 µM to 600 µM ATP. The flow cytometry profiles illustrate the P2X7-dependent shedding of CD62L from the gated CD4+ T cells after incubation with the indicated dose of ATP in mice injected 12 weeks earlier with the indicated dose of AAV-13A7-Fc vector. The indicated numbers correspond to the percentages of CD62Lhigh cells in each experimental condition after treatment with ATP. The graph bars recapitulate the data obtained in this experiment for each indicated dose of AAV-13A7-Fc vector. Figure S2. Evaluation of the blocking effect of AAV-13A7-Fc vector on the activity of P2X7 on the surface of CD8+ T cells 12 weeks after i.m. administration of different doses of AAVnano vector. AAV-13A7-Fc vector was injected i.m. with the indicated AAVnano dose and blood cells were collected 12 weeks later to evaluate the functional activity of P2X7 on the surface of CD8+ T cells after incubation with the indicated concentration of ATP ranging from 150 µM to 600 µM. The flow cytometry profiles illustrate the P2X7-dependent shedding of CD62L from the gated CD8+ T cells after incubation with the indicated dose of ATP in mice injected 12 weeks earlier with the indicated dose of AAV-13A7-Fc vector. The indicated numbers correspond to the percentages of CD62Lhigh cells in each experimental condition after treatment with ATP. The graph bars recapitulate the data obtained in this experiment for each indicated dose of AAV-13A7-Fc vector. Figure S3. Evaluation of AAVnano toxicity. Mice (n = 5) received i.m. 1011 vg of AAV coding fo [file 12951_2023_2285_MOESM1_ESM.docx]

**Additional file**

**
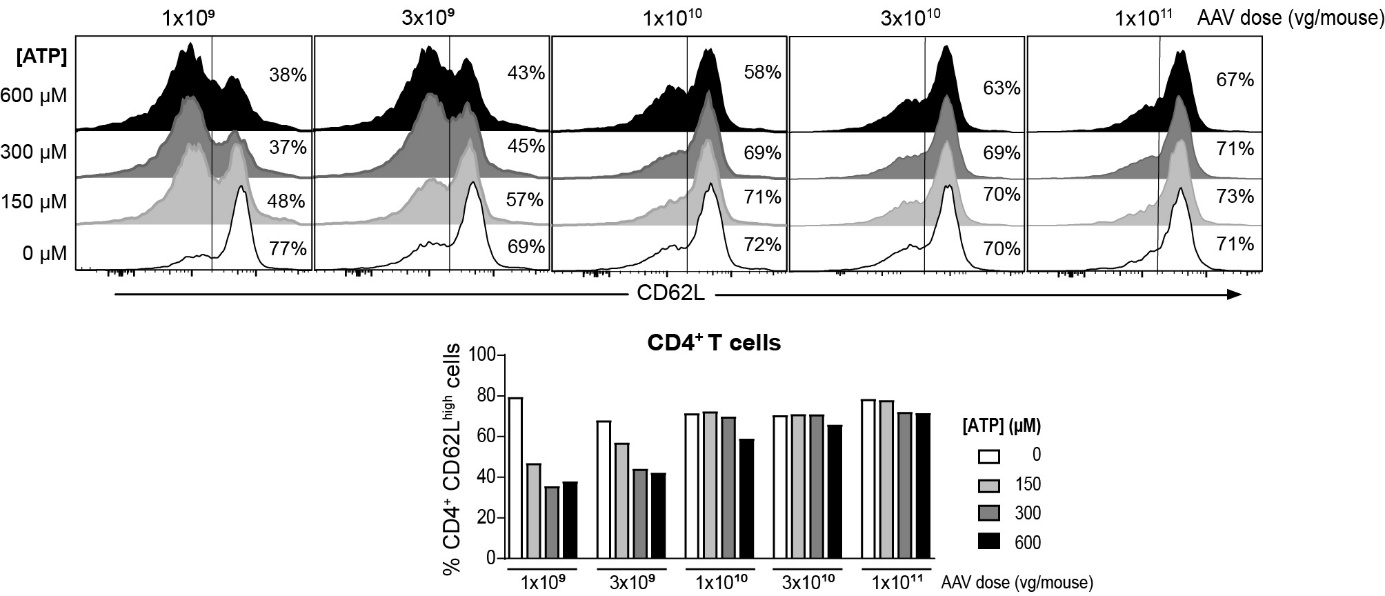
**

**Figure S1. Evaluation of the blocking effect of AAV-13A7-Fc vector on the activity of P2X7 on the surface of CD4^+^ T cells 12 weeks after i.m. administration of different doses of AAVnano vector.**

AAV-13A7-Fc vector was injected i.m. with the indicated AAVnano dose and blood cells were collected 12 weeks later to evaluate the functional activity of P2X7 on the surface of CD4^+^ T cells after incubation with the indicated dose of ATP ranging from 150 µM to 600 µM ATP. The flow cytometry profiles illustrate the P2X7-dependent shedding of CD62L from the gated CD4^+^ T cells after incubation with the indicated dose of ATP in mice injected 12 weeks earlier with the indicated dose of AAV-13A7-Fc vector. The indicated numbers correspond to the percentages of CD62L^high^ cells in each experimental condition after treatment with ATP. The graph bars recapitulate the data obtained in this experiment for each indicated dose of AAV-13A7-Fc vector.

**
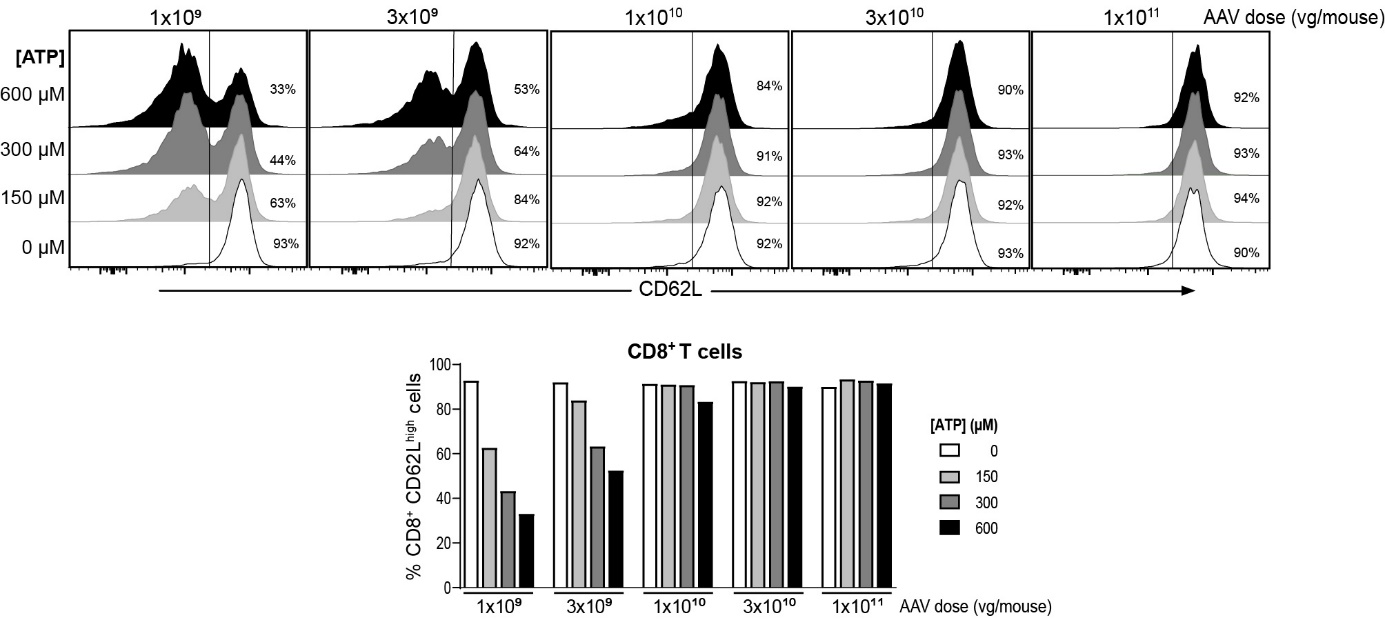
**

**Figure S2. Evaluation of the blocking effect of AAV-13A7-Fc vector on the activity of P2X7 on the surface of CD8^+^ T cells 12 weeks after i.m. administration of different doses of AAVnano vector.**

AAV-13A7-Fc vector was injected i.m. with the indicated AAVnano dose and blood cells were collected 12 weeks later to evaluate the functional activity of P2X7 on the surface of CD8^+^ T cells after incubation with the indicated concentration of ATP ranging from 150 µM to 600 µM. The flow cytometry profiles illustrate the P2X7-dependent shedding of CD62L from the gated CD8^+^ T cells after incubation with the indicated dose of ATP in mice injected 12 weeks earlier with the indicated dose of AAV-13A7-Fc vector. The indicated numbers correspond to the percentages of CD62L^high^ cells in each experimental condition after treatment with ATP. The graph bars recapitulate the data obtained in this experiment for each indicated dose of AAV-13A7-Fc vector.

**
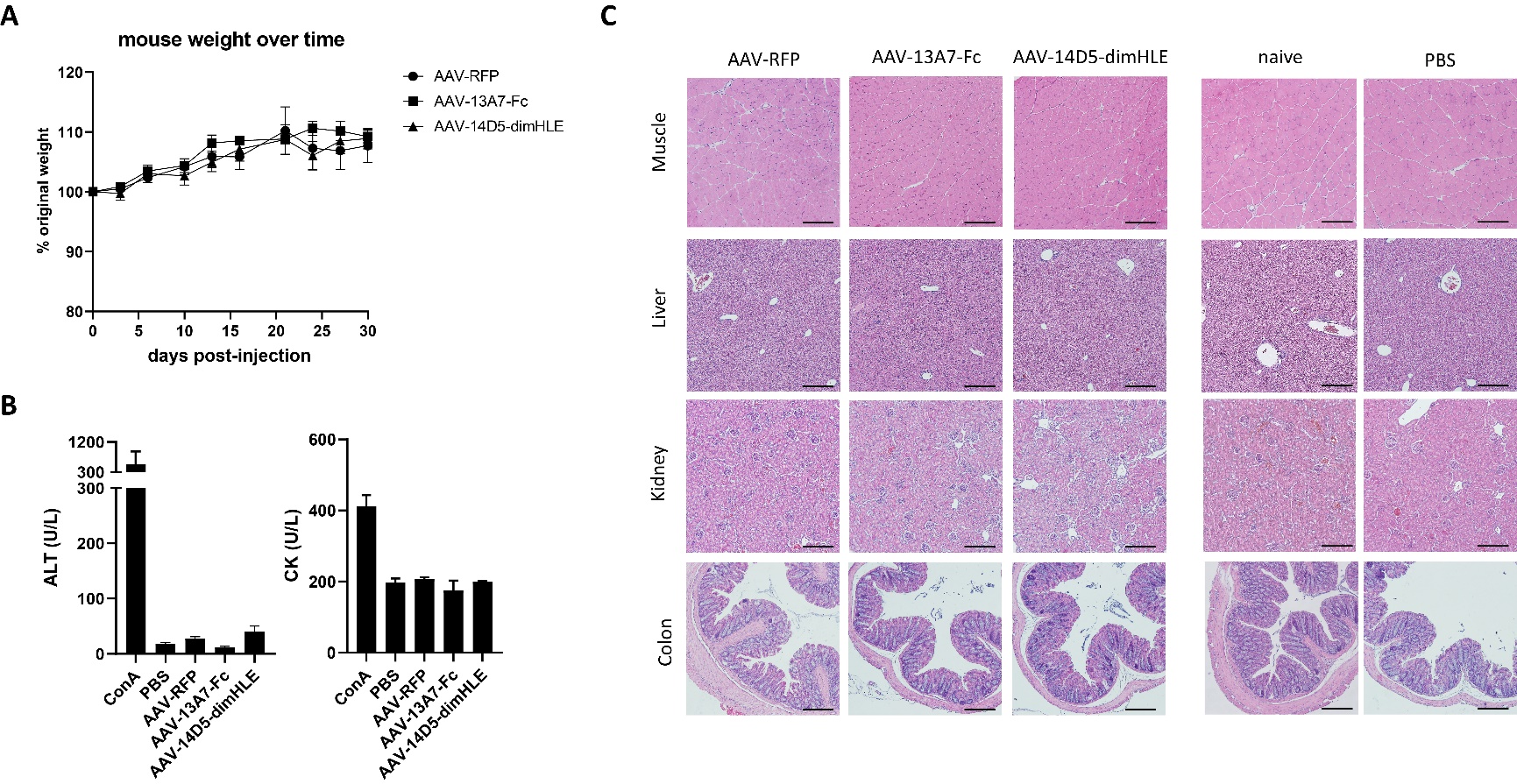
**

**Figure S3. Evaluation of AAVnano toxicity.**

Mice (n=5) received i.m. 10^11^ vg of AAV coding for RFP (AAV control), 13A7-Fc or 14D5-dimHLE. **A**. Weight follow up over time (% of original weight). **B**. Serum enzymatic activity of alanine amino transferase (ALT) and creatine kinase (CK) measured on day 30 post-injection. We used the concanavalin A (ConA) liver toxicity model as a positive control. For that, 5 mice were injected i.v. with ConA (20 mg/kg), and serum was collected 8 hours later. **C**. Representative images of muscle, liver, kidney and colon sections from AAV-injected mice stained with hematoxylin and eosin (scale bar correspond to 200 µm; n=5 mice/group). Representative images of control mice (naïve or PBS injected, n=5 mice/group) are shown on the right panels.

**
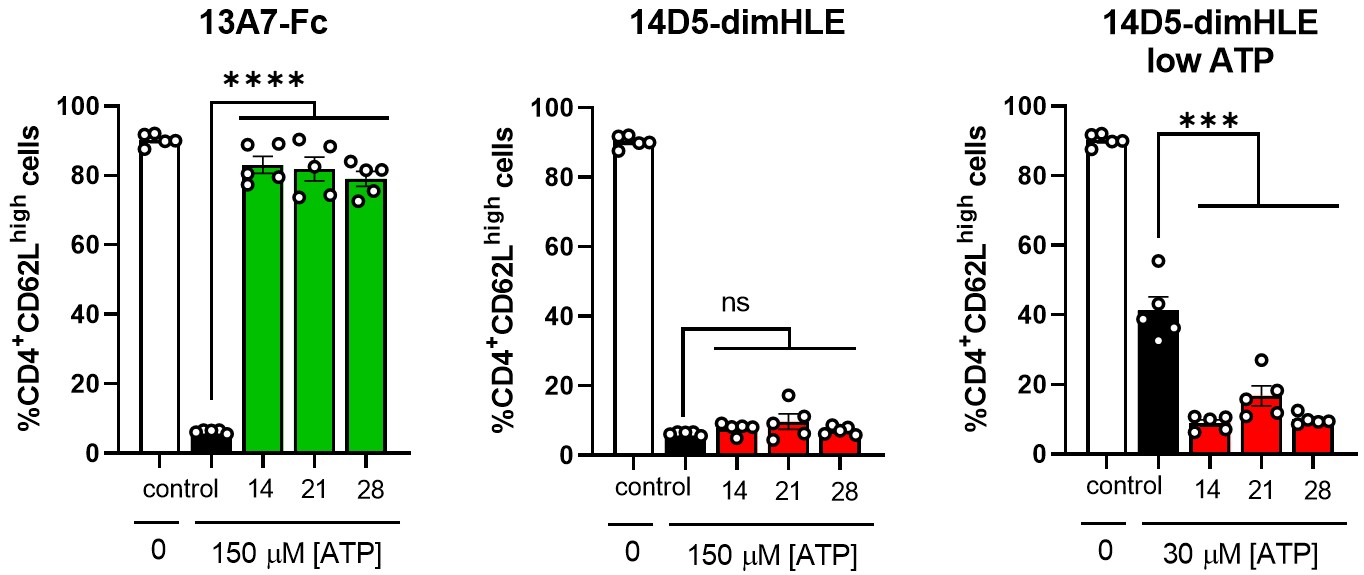
**

**Figure S4. Evaluation of the AAV-13A7-Fc blocking and of the AAV-14D5-dimHLE potentiating vectors on the activity of P2X7 overtime upon a single i.m. injection of each AAVnano vector.**

As in Figure 4, AAV-13A7-Fc or AAV-14D5-dimHLE vectors were injected i.m. at a dose of 10^11^vg/mouse and blood cells were collected at the indicated time points. Blood cells were incubated with 150 µM or 30 µM ATP, as indicated, and the percentages of CD4^+^CD62L^high^ cells were determined by flow cytometry as an evaluation of P2X7 activity on the surface of the gated CD4^+^ T cells. The first two bars in each graph correspond, respectively, to negative and positive control cells collected from control mice receiving AAV-RFP control AAV vector, treated or not with the indicated concentration of ATP. Results represent mean values ± SEM, with n=5 mice per group.

**
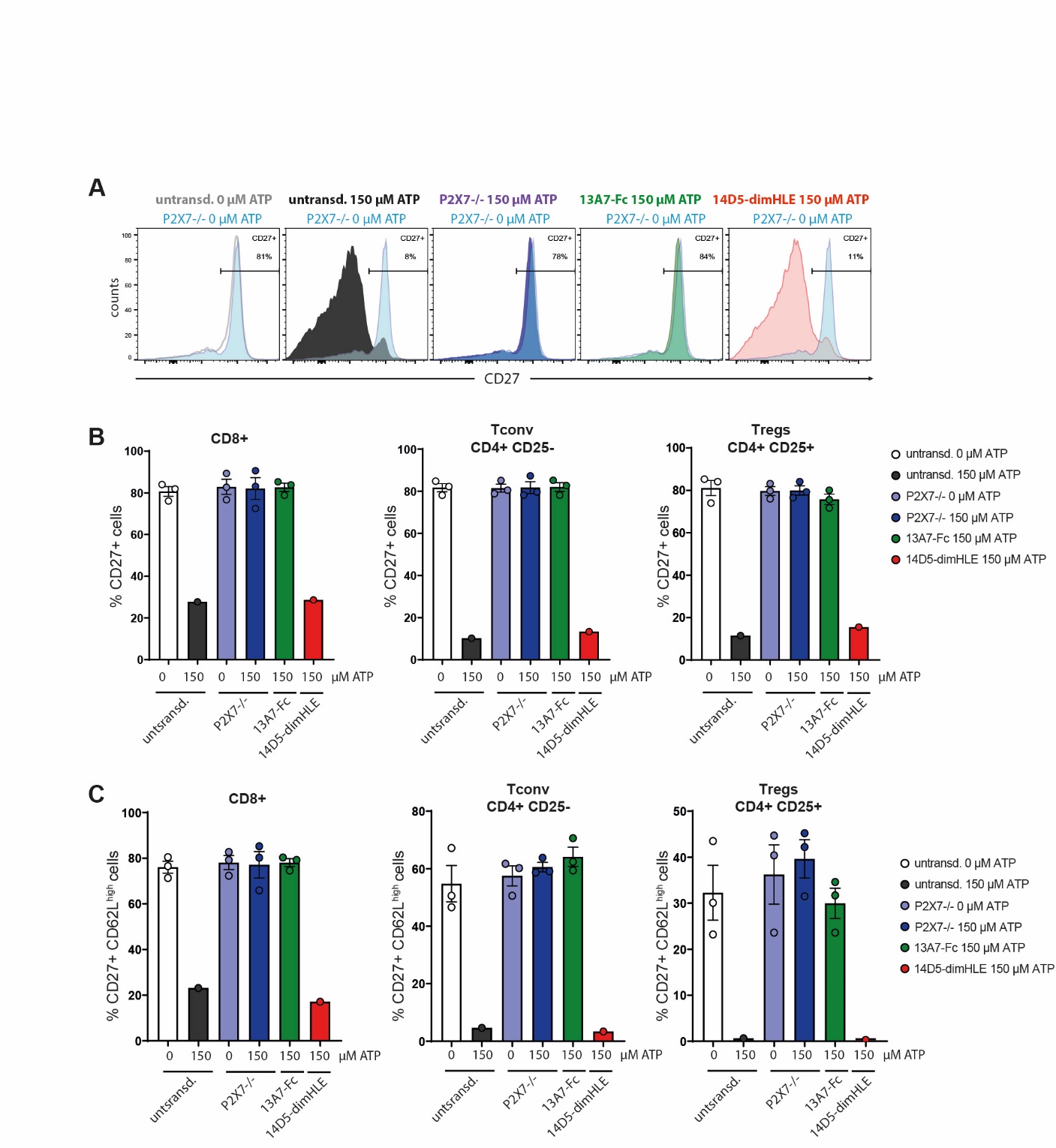
**

**Figure S5. Comparison of P2X7 activity on the surface of T cells collected from AAV-13A7-Fc injected mice or from P2X7^-/-^ mice.**

AAV-13A7-Fc vector was injected i.m. at a dose of 10^11^ vg/mouse and splenocytes were collected 120 days later to evaluate the functional activity of P2X7 on the surface of the indicated T cell subset and were compared to T cells collected from P2X7^-/-^ mice using the same experimental conditions. **A**. Histogram overlay showing expression of CD27 on the surface of CD4+ cells collected from untreated P2X7-deficient mice (green histograms) or from the WT mice that were treated as indicated (red histograms). Bar graphs showing the percentage of CD27^+^ cells (**B**), or CD27^+^CD62L^high^ (**C**) in the indicated gated T cell subset.


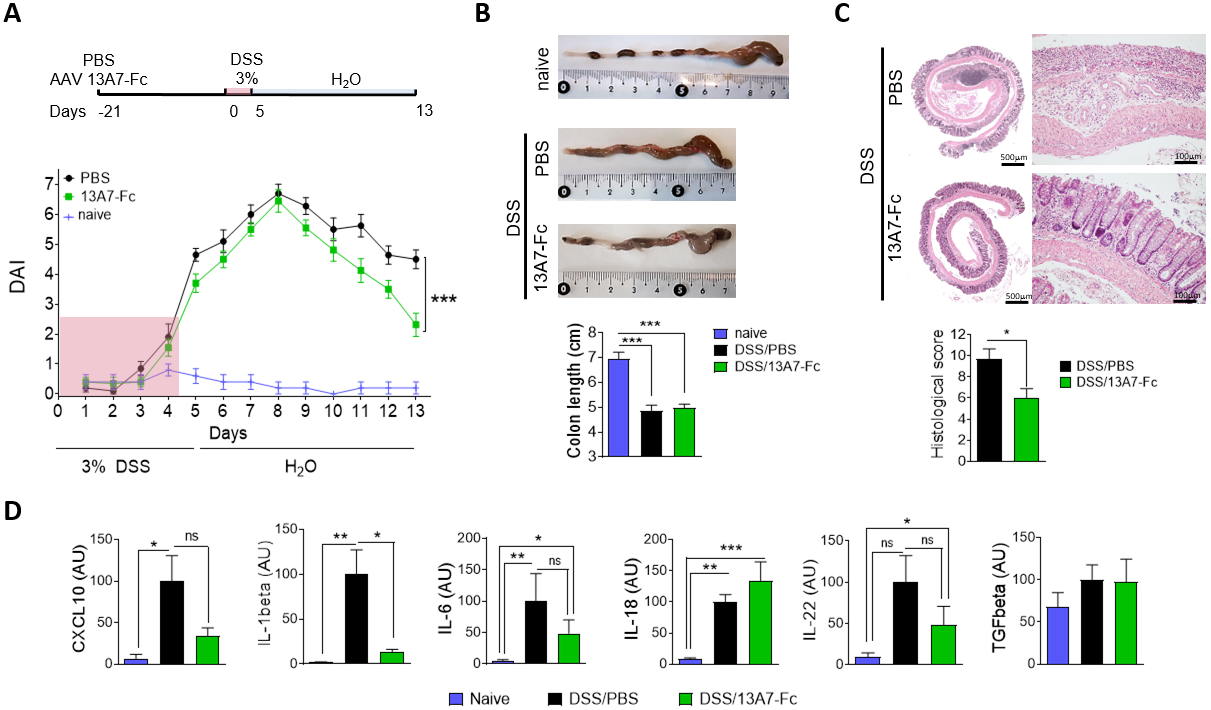


**Figure S6. AAV-13A7-Fc administration ameliorated high-dose DSS-induced colitis.**

Mice (n=10) received 10^11^ vg/mouse of AAV coding for 13A7-Fc or 14D5-dimHLE and three weeks later they were given 3 % DSS in drinking water. Mice were switched to regular water for eight additional days and colons were collected at the end of the study. **A**. Disease activity score (DAI) from 13A7-Fc and PBS-injected mice with a higher dose of DSS (3 %). **B**. Colon macroscopic appearance of naïve, untransduced and 13A7-Fc mice. **C**. Histopathological analysis of colon stained with hematoxylin and eosin staining. **D**. mRNA expression of chemokine CXCL10 and cytokines (TGF-β, IL-1β, IL-6, IL-18 and IL-22) in the colons of mice injected with AAV coding for 13A7-Fc and exposed to DSS (day 13 from the beginning of DSS treatment). Mean ± SEM are shown. For statistical comparison two-way ANOVA (**A**) or one-way ANOVA (**B** and **C**) were performed with *p<0.05 and **p<0.01.

**Table S1: List of RT-qPCR primers**

| **CCL2/MCP-1** | NM_011333 | 5’-AAAAACCTGGATCGGAACCAA-3’ | 5’-CGGGTCAACTTCACATTCAAAG-3’ |
| --- | --- | --- | --- |
| **CXCL10/IP-10** | NM_021274 | 5’-TGCTGGGTCTGAGTGGGACT-3’ | 5’-CCCTATGGCCCTCATTCTCAC-3’ |
| **CXCL9/MIG** | NM_008599 | 5′-TCTGCCATGAAGTCCGCTG-3’ | 5′-CAGGAGCATCGTGCATTCCT-3’ |
| **IFNγ** | NM_008337 | 5’-TGCTGATGGGAGGAGATGTCT-3’ | 5’-TTTCTTTCAGGGACAGCCTGTT-3’ |
| **IL-17A** | NM_010552 | 5′-GGAGAGCTTCATCTGTGTCTCTG-3′ | 5′-TTGGCCTCAGTGTTGGACA-3′ |
| **IL-18** | NM_008360.2 | 5’-GACTCTTGCGTCAACTTCAAGG-3’ | 5’-CAGGCTGTCTTTTGTCAACGA-3’ |
| **IL-1beta** | NM_008361.4 | 5′-TGGACCTTCCAGGATGAGGACA-3′ | 5′-GTTCATCTCGGAGCCTGTAGTG-3′ |
| **IL-22** | NM_016971.2 | 5’-TGACGACCAGAACATCCAGA-3’ | 5’-AGCTTCTTCTCGCTCAGACG-3’ |
| **IL-6** | NM_010551 | 5’-TTCCATCCAGTTGCCTTCTTG-3’ | 5’-TTGGGAGTGGTATCCTCTGTGA-3’ |
| **Eef2** | NM_007907.2 | 5’-AAGCTGATCGAGAAGCTGG-3’ | 5’-CCCCTCGTATAGCAGCTCAC-3’ |
